# A hybrid ML-PBPK digital twin framework for clinically interpretable readmission risk and drug exposure stratification in diabetes

**DOI:** 10.3389/fdgth.2026.1867505

**Published:** 2026-09-01

**Authors:** Nihaal Ahmed.K, Jafar Ali Ibrahim Syed Masood

**Affiliations:** 1 School of Computer Science and Engineering, Vellore Institute of Technology, Vellore, Tamil Nadu, India; 2 Department of Quantum AI, School of Computer Science and Engineering, Vellore Institute of Technology, Vellore, Tamil Nadu, India

**Keywords:** clinical decision support, diabetes mellitus, digital twin, drug exposure modeling, PBPK simulation, readmission prediction, XG boost

## Abstract

The prediction of whether a diabetic patient will return to the hospital within 30 days is a very difficult clinical situation to navigate. Predicting the chance of a 30- day readmission depends on a wide range of patient-specific risk factors and the variability in how individual patients respond to medication prescribed for them. Traditional machine-learning models are capable of finding patterns in electronic medical records but are typically unable to provide a clear picture of the physiological interactions between the patient and their prescribed drug(s). One way to predict the likelihood of diabetes-related rehospitalization is using a combination of machine-learning risk factors and physiologically based pharmacokinetics (PBPK). An optimized version of the XG Boost machine-learning algorithm was trained on a large in-patient dataset (approximately 100,000 patient encounters) and achieved a predictive model with an area under the receiver operating characteristic curve (AUC) of 0.68 with a recall of 0.60 at clinically meaningful cut-offs. These findings are similar to what has been previously reported in the literature (0.60–0.70) for diabetes-related rehospitalization risk and support the utility of the XG Boost algorithm as a reliable clinical screening tool. Concurrently, multiple PBPK simulations were run to assess the effects chronic renal or hepatic impairments have on PBPK; results show renal impairment results in the highest systemic drug exposures. Predictive risk and simulated exposure will be integrated to yield a patient-specific risk/exposure phenotype and enable clinically meaningful stratification into actionable subgroups. Through this new method, it becomes possible to discern pharmacological vs. non-pharmacological contributors to readmission risk, thus facilitating targeted intervention strategies such as dose modulation, enhanced monitoring, and coordinated care. This work's value lies not in improving prediction accuracy by just a few points, but rather in a new approach to converting static risk prediction into a clinically actionable decision support system based on physiology. The proposed Digital Twin framework offers a scalable path to personalized post-discharge patient management and provides the basis for furthering the implementation of precision medicine in the field of digital health.

## 1 Introduction

### 1.1 Background and clinical importance of diabetes readmission

Hospital readmission in diabetes patients continues to pose a significant burden on global public health, leading to higher healthcare use, economic cost and negative patient outcomes. Diabetes is a chronic and debilitating, life-threatening condition, with a high rate of hospitalization for its myriad complications such as cardiovascular events (1), renal injury (2), infections (3) and metabolic instability (4). Although inpatient management has been improving, almost 10% of diabetic patients will have unplanned readmission within the 30-day post discharge period-the aspiration gap that still exist for the post discharge care and self-management support. Not surprisingly, all these early readmissions are related to the higher mortality and a greater risk of persistent complications in the long-term, highlighting the importance of identifying remotely those patients to be at high-risk with time to implement appropriate interventions. Useful prediction of readmission risk will facilitate healthcare providers in delivering interventions, personalized treatment plans and health service enhancement. This aligns well with the United Nations SDG3 (Good Health and Well-being goal) that aims to optimize care of chronic disease patients with the new paradigm of predictive and personalized care.

### 1.2 Limitations of existing readmission prediction models

At present, predictive analytics has been widely used in the healthcare field; however, current readmission prediction models are less efficient for diabetic patients. “echo” Traditional statistical methods like logistic regression and initial machine learning models— such as support vector machines, decision trees, basic ensembles — tend to produce low predictive accuracy or are not sensitive enough for clinical use (1–3). The majority of models consider patient populations as one patient not taking into account physiological differences and variations in drug digesting. Moreover, those models generally only utilize structured EHR data, which is unable to entirely represent the complex interaction of clinical, behavioral and pharmacological elements that affect readmission. Another significant constraint is the lack of transparency as most models operate similar to “black boxes,” resulting in clinicians having difficulty understanding how predictions are made. Poor generalizability, lack of adequate calibration and poor clinical interpretability have all combined to stall the adoption of such models into standard care pathways. Thus, new modelling methodologies are required to address these deficiencies, integrating physiological knowledge and depicting the patient state in a more comprehensive manner.

### 1.3 Physiological variability and its impact o drug response

Large population-derived variability in the baseline physiology is a dominant factor affecting treatment responses of diabetic patients that has been neglected by traditional readmission prediction models. Renal insufficiency, liver failure, changes in cardiac output and shift of body composition may greatly influence the PK and PD of drugs. For instance, impairment of kidney function decreases the elimination of several widely used diabetes drugs, leading to higher systemic levels and subsequently increased risk for toxic reactions. Quantitatively and qualitatively, hepatic insufficiency can change drug metabolism, resulting in erratic fluctuations of plasma concentration. Differential body weights and their associated volume of distribution can also affect absorption and bioavailability. It is expected to contribute to increase the medication efficacy, the stabilization of glucose level and the total patient recuperation during the vulnerable period post discharge. These variations, ignored by the models could mislabel patients with high risk. The recognition of this physiological heterogeneity is necessary for accurate, personalized, and clinically relevant predictors that match the reality of pathophysiological processes.

### 1.4 Role of PBPK modeling in personalized therapy

Physiologically-Based Pharmacokinetic (PBPK) modeling is an important approach for new drug development and optimal therapeutics. PBPK modeling is based on detailed anatomy, physiology, and biochemistry in order to predict drug absorption, distribution, metabolism, and excretion (ADME) among different populations. These models enable investigation of drug exposure in populations undergoing renal or hepatic dysfunction, children and geriatrics, as well as physiological conditions such as abnormal body by researchers and clinicians. PBPK calculations generate concentration–time courses predicting systemic drug exposure, which clinicians can use to tailor dosing regimen according to the results, and with regard to an expected side effect profile. Although PBPK modeling is particularly advantageous to grasp biological mechanisms that govern drug clearance, it lacks the capacity of integrating larger aspects of clinical pharmacology, such as concurrent morbidities and treatment history and broader healthcare/ socioeconomic variables. Thus, PBPK models are insufficient to predict real-world outcomes such as early hospital readmission. Inclusion of PBPK insight in data-driven, predictive modeling affords an opportunity to develop holistic and personalized clinical decision support tools.

### 1.5 Need for integrating mechanistic and data driven framework

With the growing recognition of the complexity involved in chronic disease care, clear limitations have emerged in using either exclusively statistical or exclusively mechanistic methods when devising models. Machine learning is good at detecting non-linear patterns in high-dimensional clinical data, whereas PBPK models have a mechanistic basis and physiological interpretation. Yet, these two paradigms have not often met while studying readmissions in the clinical literature. Hybrid frameworks are missing, which is a significant gap in the field, given that integration between ML and PBPK could lead to more reliable, personalized and physiological realistic predictions. Recent developments in Digital Twin strategies have accentuated the demand for virtual patient models, including the combined biological and clinical performance. Despite increasing popularity, DTS systems are currently mostly conceptual and do not integrate real-world clinical reports with mechanistic drug exposure simulation. A combined Integrated ML–PBPK Framework could serve to narrow this gap, achieving enhanced predictive abilities and better interpretability as well as richer physiological insight personalized for individual patient attributes.

### 1.6 Research objective and hypothesis

This work's primary goal is to propose a physiologically contextualized Digital Twin framework that integrates machine-learning based prediction of readmission risk with PBPK modeling of individual drug exposure profiles. In contrast with purely performance-optimizing prediction models, the proposed framework will characterize the risk-exposure relationship at an individual level, thus enabling clinically interpretative individual risk-exposure characterization of readmission risk. Our hypothesis is that the integration of machine-learning risk prediction and PBPK exposure estimates can enable a more physiologically contextualized and clinically interpretable individual prediction beyond typical EHR-based models, with a comparable risk-predictive performance.

Within this work Digital Twin refers to patient specific computation models combining predictive and mechanistic models for personalised risk evaluation and scenario testing. The developed framework has to be understood as a clinical Digital Twin proof-of-concept, rather than an real-time fully synchronized Digital Twin.

### 1.7 Novelty and scientific contribution of this study

This paper proposes a hybrid Digital Twin model, which combines the machine-learn-based readmission predictive model with the PBPK-based drug exposure simulation for diabetic patients (Table 1). In contrast to currently available models which are based on EHR records, the present approach is capable of reflecting physiological variability through simulation of drug concentration-time profiles in healthy, renal- and hepatic-impaired, as well as low (high) body weight individuals. The machine learning section: We have developed an optimized XGBoost model based on the anonymized full training set of more than 100,000 hospital encounters for better discriminatory performance (AUC = 0.69) and high sensitivity (recall = 0.60) in clinical meaningful thresholds. SHAP values also contribute to the model's interpretability, disclosing clinically intuitive predictors like previous inpatient visit, discharge destination and emergency encounters. The PBPK model consistently displays significant alterations in the systemic exposure of the drug based on physiological state, with renal insufficiency and hepatic insufficiency exhibiting 40%–50% higher AUC relative to a normal physiologic state. By associating ML-derived risk scores with mechanistic drug exposure metrics, this study unveils a novel and clinically relevant risk–exposure phenotype. To the best of our knowledge, this is among the first integrated ML–PBPK Digital Twin models applied to diabetes readmission prediction, and facilitated emerging global innovation endeavours coordinated with SDG 9: Industry, Innovation, and Infrastructure; and more equitable healthcare (SDG 10). Unlike prior Digital Twin or hybrid ML–PBPK studies that focus on drug response or dose optimization alone, this work introduces a clinical outcome–oriented Digital Twin in which PBPK simulations are not used as predictive features but as physiological contextualizes of machine-learning risk estimates. This distinction is critical: the proposed framework does not aim to improve discrimination metrics alone, but to transform readmission prediction into an interpretable risk–exposure phenotype, enabling clinically actionable insights. To the best of our knowledge, no prior study has operationalized PBPK simulations within a Digital Twin architecture for hospital readmission prediction in diabetes, nor demonstrated how mechanistic exposure variability can be used to contextualize ML-derived risk for post-discharge decision support.

**Table 1:** Comparative summary of existing digital twin frameworks in healthcare and diabetes management.

Area	What existing studies do	Identified gap in literature	How our proposed hybrid digital twin framework addresses the gap
1. Readmission Prediction in Diabetes (Section 2.1)	Uses logistic regression, regression trees, SVM, random forests, and deep learning to predict 30-day readmission (AUC typically 0.60–0.70). Mostly relies on demographic and administrative features.	Existing models lack clinical precision, have poor recall, and ignore physiological or drug-response factors. They also do not personalize predictions.	Existing models lack clinical precision, have poor recall, and ignore physiological or drug-response factors. They also do not personalize predictions.
2. ML Approaches Using EHR Data (Section 2.2)	ML models capture nonlinear relationships but rely heavily on retrospective EHR data and statistical patterns.	ML-only models suffer from low interpretability, dataset bias, and a lack of biological grounding. They ignore pharmacokinetics and drug variability.	Adds PBPK-based individualized drug exposure metrics into ML predictions to create biologically informed risk modeling with mechanistic interpretability.
3. Limitations of ML in Clinical Settings (Section 2.3)	ML models often lack generalizability, transparency, and the ability to incorporate inter-individual physiological differences (renal function, hepatic clearance, weight).	No current ML models include drug metabolism, organ impairment, or physiological heterogeneity in readmission prediction.	Uses PBPK simulations to account for renal impairment, hepatic dysfunction, and body-weight variability, embedding physiological realism into the prediction workflow.
4. PBPK Modeling for Diabetes Therapy (Section 2.4)	PBPK models estimate ADME processes, simulate drug exposure, and evaluate dosing strategies across physiological states.	PBPK models alone cannot predict clinical outcomes like readmission because they lack integration with EHR variables. They operate independently of real-world data.	Couples PBPK exposure outputs (AUC) with ML-predicted risk scores, forming the first PBPK-informed digital twin for diabetes readmission.
5. Hybrid Mechanistic–ML Models (Section 2.5)	Some studies combine ML with mechanistic models for dosing, toxicity, or disease progression, but these are narrow in scope and limited to specific drugs or small datasets.	No prior study has applied a hybrid ML–PBPK model to large-scale hospital readmission prediction, especially for diabetes. No integration of mechanistic exposure + EHR-driven prediction in a single pipeline.	Introduces a unified hybrid architecture linking EHR-based ML risk prediction with physiologically simulated drug exposure in a Digital Twin environment. First demonstration on a large real-world diabetes dataset.
6. Digital Twin Frameworks in Healthcare (Section 2.6)	Digital Twins exist in cardiology, oncology, and ICU monitoring; they use ML and sensor data but rarely include pharmacokinetics or large-scale EHR readmission outcomes.	No digital twin for diabetes that integrates PBPK modeling, readmission risk prediction, and EHR-derived features. Existing DTs are limited to glucose simulations or general prediction tasks.	Builds the first Digital Twin for diabetes readmission, combining: ML risk predictions PBPK exposure simulations Phenotype mapping (2D/3D). Provides actionable, interpretable, and personalized insights.

### 1.8 Rationale for the proposed digital twin framework

The notion of a Digital Twin clinic representation of an individual (4), which would bring together real-world data with mechanistic models and predictive analytics, provides a valuable frame for precision medicine. In the natural history of diabetes, in which clinical history and drug-induced physiological responses determine outcomes, a Digital Twin strategy is more comprehensive in representing patient risk. The ML-predicted readmission risk, together with PBPK-derived DAE provide an additional dimension of patient-specific vulnerability during the post-discharge period. This two-level modeling approach allows for better personalized follow-up planning, a more accurate medication management and moreover an early recognition of patients who need to be monitored more intensively. In addition, coupling a mechanistic and data-driven understanding increases model transparency and is in line with regulator expectations for interpretable decision support tools. Accordingly, the proposed platform serves as the scientific and practical basis to link predictive analytics with personalized drug response in diabetes care. In this research, we present a Digital Twin as a patient's virtual model, which includes data-driven risk prediction with mechanical physiological simulation, and which in turn enables personal scenario analysis instead of a static risk score.

### 1.9 Overview of study methodology

The methodology of this work consists of the multi-stage pipeline that begins with extensive preprocessing of the Diabetes 130-US Hospitals dataset; not only imputation but also encoding, scaling and addressing the class imbalance. Feature engineering was performed to enrich the representation of clinical, demographic and drug-related features. An optimized XGBoost model was trained with stratified splits for both training and validation, before testing the model on a random separate test set, then calibration of its threshold values, after which SHAP-based interpretability analysis was conducted. Concomitantly, PBPK models were constructed for various physiologic conditions to estimate the drug concentration–time profile and the systemic exposure in the form of AUC. The ultimate Digital Twin model combines ML-predicted risks with PBPK-derived drug exposure metrics to build a combined risk–exposure profile for each patient. This methodological pipeline can be used to give a thorough evaluation of both clinical and physiological correlates of readmission risk, laying the groundwork for a hybrid precision-medicine model.

### 1.10 Alignment with sustainable development goals (SDGs)

The developed Digital Twin demonstrates strong relevance to several of the United Nations SDGs, indicating its applicability beyond clinical medicine. More broadly, this research contributes to SDG 3 (Good Health and Well-being) by facilitating early risk stratification in diabetic patients who are high-risk for readmission, thus enhancing continuity of care with goal-directed post-discharge planning. The machine learning and PBPK simulation integration is relevant for SDG 9 (Industry, Innovation and Infrastructure) as it can stimulate the use of advanced methods in digital health and computational innovation in a clinical context. In addition, bringing in sources of physiological variation such as renal impairment or hepatic impairment (which are more frequent in disadvantaged populations), the framework indirectly addresses SDG 10 (Reduced inequalities). This framework enables fairer treatment delivery, and has the potential of providing personalized prediction and therapy advice to a more inclusive set of patients. With these contributions, it is hoped that the study will continue to explore science and offer food for thought to advance global health transformation and sustainable medical innovation.

### 1.11 Study contribution overview

In this paper we present the first clinically interpretable Digital Twin framework that simultaneously combines machine learning driven prediction of readmission risk with PBPK model based drug exposure modeling. The combination of the two models allows for simultaneous reasoning over clinical risk and physiological variance providing a patient-specific risk-exposure phenotype. As compared to static predictions made by traditional models, the framework is amenable to clinically actionable decisions by delineating the pharmacologic and non-pharmacologic drivers of readmission risk. This integrated viewpoint enhances interpretability, allows for individualized intervention strategies and moves toward decision-support systems for the digital health environment of tomorrow.

### 1.12 Manuscript layout

The remainder of the paper is organized to present the hybrid Digital Twin framework in a detailed and systematic fashion. Section 2 includes a broad literature review, including current diabetes readmission models, PBPK applications and state of the art hybrid mechanistic-machine learning strategies. Section 3 describes the method and dataset preprocessing and model development which include PBPK simulation design and amalgamation to develop the Digital Twin. Experimental results. Section 4 subsequently reports the experimental results in term of model performance, interpretability analysis, simulated prediction (PBPK derived) and synthesized risk-exposure phenotype. Section 5 discusses widely about interpretation of the results in relation to previous literature, highlighting the scientific and clinical contributions of the hybrid framework. The conclusion, Section 6, provides a summary of the key outcomes and findings, limitation and future directions for this work toward Digital Twin technologies in clinical diabetes management. It should be noticed that the sections follow the conventions used for publication in computational and biomedical science to ensure accuracy, clarity and consistence.

## 2 Literature review

In this section, we will review literature of digital twin framework (DTF), machine learning for healthcare applications and Physiologically-based Pharmacokinetic Modelling with their state-of-the-art and limitations in diabetes management. We moreover examine how they have been used to predict disease progression, optimize therapeutic strategies and improve personalized care in particular—revealing substantial shortcomings that this hybrid methodology developed herein is meant to tackle (5, 6). The use of the digital twin, specifically the human twin model for personalized diabetes intervention, enabled modeling with diverse patient-specific data to create a mirrored image of an object and simulate the synthesis and control of disorders such as Type 2 Diabetes in the elderly (7). This real-time monitoring and analysis ability permits both disease progression simulation and care plan automation (6, 8). These digital twins integrate machine learning with multiomic data and knowledge graphs to provide a comprehensive management strategy for type 2 diabetes (6), serving as a next generation of personalized-precision-systems medicine paradigm (7).

### 2.1 Diabetes readmission and the need for accurate prediction models

One of the most prevalent obstacles to overcome is readmission into the hospital among patients with diabetes, due to its chronic and multifactorial nature. Multiple studies have suggested unplanned readmissions within 30 days correlated with suboptimal metabolic control, complications, and inadequate post discharge care (9). Conventional risk assessment approaches are substantially clinical or based on rules, and they provide low precision (2, 3). As medical databases have grown, interest has turned to (computational) models able to detect patterns that might be overlooked by traditional investigation. Yet, performance of predictive power reported across studies is quite disparate, with a lot of models being accurate only at a fair level and too low a recall value to be reliable for use in the clinic (10, 11). This underscores an urgent need for such advanced context-aware predictive models that combine multiple types of data streams and physiological wisdom to enhance accuracy as well as clinical relevance in diabetes management (6, 12). For example, using the digital twin technology based on a patient's past and present health measurements may offer new opportunities for personalized predictive modelling, especially in diseases [such as Type 2 Diabetes (T2D)] where there is a need to integrate short-term observations with long-term history of condition/s (12). On-the-fly reconfigurable digital twins, which include health knowledge graphs and real-time data, will provide a dynamic method to understand individual patient trajectories and provide personalized interventions (13, 14). This allows to simulate possible health problems over time and apply personalized preventive interventions beforehand, by minimizing drug dosage and treatment effects (15). By combining AI and digital twins, a more complete personalized care can be conceived, going beyond general medical recipes to individual patient realistic plans. These constraints imply that early readmission prediction should rely less on administrative predictors in favor of stronger and physiologically interpretable models (6).

### 2.2 Machine learning approaches for readmission prediction

There have been many uses of machine learning (ML) techniques in predicting readmission in diabetes, among which are logistic regression, support vector machine (SVM), random forest, and neural networks or gradient boosting models (3, 16). These methods use high-dimensional hospital data to capture non-linear relationships between patient factors and readmission outcomes (1, 2) Although some reports have shown superior results in comparison to conventional methodologies, the vast majority of ML classifiers consistently generate AUCs between 0.60–0.70 and recall values generally below clinically acceptable levels (17, 18). Furthermore, predictive characteristics such as previous use, co-morbidity load or medication changes are applied without further consideration of the underlying pharmacological or physiological mechanisms (19, 20). Due to a focus on retrospective EHR data, such models have limited biological interpretability and tend to be subject to dataset-specific biases (21). This implies that ML-only strategies are incomplete for real-world clinical translation, despite the promise they hold. A hybrid system which combines mechanistic physiological modeling and data-driven machine learning approaches may provide a more interpretable and robust model of readmission prediction in diabetes. This integration may potentially result in more advanced digital twins that not only are predictive but also prescriptive by also providing individualized interventions according to real-time physiological responses (22). For example, long short-term memory model-based recurrent neural networks have shown encouraging results in predicting 30-day readmissions for patients diagnosed with diabetes when using a large amount of longitudinal data from prior visits (3).

### 2.3 Limitations of existing ML models in clinical settings

One of the major limitations of existing ML models is the lack of consideration for inter-individual physiological differences. Medication metabolism, organ system function and body composition, all of which are important determinants of therapeutic stability are seldom included (23, 24). In addition, ML models trained from imbalanced samples tend to focus more on accuracy than sensitivity, which will result in a systematic under-spotting of high-risk patients. Model outputs routinely fail to provide clear decision pathways, thus undermining the confidence of clinicians who demand interpretable decision-support systems (25). An additional problem is the ability of an ML model to generalize across institutions since disparities in coding practices, treatment strategies and patient population may limit external validity (26). These observations suggest that ML-alone frameworks are not likely to capture the complexity of readmission risk and motivate the development of hybrid models that combine mechanistic understanding with data-driven inference. For instance, although some works are able to achieve high recall, their computational complexity and the expertise of personnel can be a barrier for practical utilization (18).

### 2.4 Physiologically-based pharmacokinetic (PBPK) modelig in diabetes therapy

Physiologically-Based Pharmacokinetic (PBPK) model is a structured mechanistic embodiment of drug ADME in various organs (27, 28). These models include physiological-parameters such as organ blood flow, tissue volumes, enzyme activity and renal clearance that can be used to simulate drug exposure in different states of the body (29, 30). PBPK approaches have been widely employed in pharmacology to assess dosing regimens for patients with renal or hepatic impairments, changes in body weight, or concurrent conditions (31, 32). They provide a quantitative assessment of concentration–time profiles and systemic exposure parameters, such as the AUC. However, PBPK models do not account for other clinical datasets, which may be broader and include hospital utilization, comorbidities or sociodemographic factors that actually determine real-world outcomes such as readmission (33–36). Accordingly, PBPK modeling is a mechanistic tool issuing from direct pharmacology and is not able to predict clinical endpoints without being combined with data-driven approaches. This discrepancy underscores the need for hybrid modeling strategies, in which PBPK models are combined with machine learning approaches to integrate real-world clinical data and anticipate more complex outcomes, such as re-admittance rates (37). This cross-feeding can potentially result in the realization of advanced digital twins which are predictive as well as prescriptive to provide a personalized set of corrective actions based on constant monitoring and real-time physiological feedback responses, thereby raising clinical significance and interpretability that such models will have (38).

### 2.5 Hybrid mechanistic-ML approaches and current gaps

New model training approaches. There has been discussion to combine mechanistic and machine learning models as an approach for increased interpretability and prediction performance in clinical applications (39–41). Such hybrid models can iteratively leverage the power of ML-based pattern recognition and multi-dimensional data fitting, while being grounded in physiology through the provision of PBPK simulation (42, 43). Finally, despite growing popularity, the number of published studies involving hybrid ML–PBPK or ML–mechanistic approaches is small, and most are oriented to drug response prediction or dose individualization (rather than readmission or longitudinal care outcomes) (42, 44, 45). To the best of our knowledge, there have been few (if any) studies using a hybrid model in large hospitals, and it is believed that literature integrating personalized drug exposure simulation with diabetes readmission prediction remains extremely limited. This gap highlights the necessity for holistic frameworks that integrate clinical and predictive modeling data, together with pharmacokinetic mechanistic understanding within an analytic framework. Filling this gap will necessitate advanced hybrid models, which can simultaneously learn from diverse sources of data, spanning molecular-level interactions to population-level clinical outcomes (46). This would enable more accurate preclinical drug exposure prediction by integrating machine learning and mechanistic models.

### 2.6 Digital twin framework in healthcare

The development of digital twin technologies is a promising solution to form computational models for individual patients and to merge real-world data streams with predictive, as well as simulation-based models (6, 47, 48). Digital Twins have been used in cardiology, oncology, surgical planning and for monitoring of intensive care patients, proving their worth in scenario modeling, disease progression modeling and optimization of personalized treatments (49, 50). Such advanced pipelines, however, require powerful approaches to incorporate various kinds of data, ranging from genetic data to real-time sensor readings of physiology, to help create comprehensive and dynamic representations of patients. To fully exploit the power of multi-modal patient data in precision medicine, next-generation digital twins should bring together statistical modeling and machine learning with systems biology/quantitative systems pharmacology models (14). This integration is essential to overcoming the current limitations in predictive accuracy and clinical relevance of most digital twin systems which commonly use “black-box” predictive models (51). There is, however, widely disparate maturity of applications in the digital twin across clinical settings. Current digital twins are limited in scope, and few of them have combined EHR-based predictive analytics with physiological drug exposure simulation and even less toward chronic metabolic disorders, such as diabetes (4, 6). This research calls for the creation of new digital twin paradigms integrating predictive analytics from electronic health records with physiologically based pharmacokinetic models to improve personal care in conditions such as diabetes (6, 52). However, instantiating digital twin models frequently suffers from limited data input ranges and simplified physiology or insufficient incorporation of pharmacokinetic/dynamic information (7, 53). Hence, we proposed the construction of a special Drug Digital Twin for the prediction of diabetes readmission, which involves ML-based risk scores and PBPK-based exposure metrics. This systems-oriented strategy will allow clearer, more actionable lessons to personalize medicine and to optimize the clinical potential of “big data,” via harmonious interactions between data-driven and mechanistic modelling paradigms. This fills an important void in the current digital twin and diabetes analytics literature (6, 52).

### 2.7 Gap analysis and novelty in our proposed work

While contributions of digital twin, machine learning models and PBPK simulation are individually substantial, we have identified no comprehensive framework in the literature to unify these components for prediction real-world clinical outcomes such as readmission risk of diabetes. Current approaches have limitations, including low interpretability (in ML-only models), lack of clinical endpoints (in PBPK-only models) or restricted domain scope (the currently available digital twins) or the utilization used in specific tasks. To the best of our knowledge, this study is among the first to develop an integrated ML–PBPK Digital Twin framework effectively designed for diabetes readmission prediction using both physiological variability in drug-response and clinical EHR-based risk factors.

Despite significant advancements in machine learning–based prediction models and physiologically based pharmacokinetic (PBPK) modeling, existing approaches remain largely fragmented in their application to clinical outcomes such as hospital readmission. Machine learning models effectively capture patterns from electronic health records but lack physiological interpretability, while PBPK models provide physiological contextualization into drug exposure but do not account for broader clinical and behavioral determinants. To the best of our knowledge, no unified framework currently integrates both ML-based readmission risk prediction and PBPK-based drug exposure modeling within a single analytical pipeline for diabetes care. Here, we will overview the literature regarding the digital twin framework (DTF), machine learning on the healthcare and the physiologically-based pharmacokinetic modelling, state-of-the art and limits in diabetes management.

Table Overview of current Digital Twin frameworks for healthcare and diabetes shows the approach and clinical usage of some existing digital twin frameworks for healthcare and diabetes. The summary clearly indicates that majority of frameworks use either prediction based on data or based on mechanisms and that there is a gap of integrated framework for analysis of both the risk and drug exposure.

## 3 Methodology

### 3.1 Dataset description

For this study we used data from the Diabetes 130-US Hospitals dataset, a publicly available dataset which consisted of 101,766 inpatient encounters across 130 hospitals across the US over the years 1999–2008. A discharge record constitutes an individual hospital visit by a patient diagnosed with diabetes. It contains data regarding demographics, medical history, laboratory production use and treatment data (including medication use) for that patient's hospital visit as well as the diagnoses and procedures that were used to manage the individual diagnoses. The dependent variable was defined as 30-day readmission; which was also dichotomized. Records lacking demographic data or lacking clear discharge status were removed to maximize analytic validity, resulting in a cohort of roughly 81 K visits. The Diabetes 130-US Hospitals dataset represents one of the largest publicly available, standardized databases for inpatient diabetes outcome research.

### 3.2 Data preprocessing

All pre-processing was carried out in Python using available scientific packages (e.g., NumPy 1.24, pandas, scikit-learn). Numerical variables with missing entries were imputed with median imputation to avoid too large distributional shifts, and a categorical variable was reverted back to the most frequent category. For categorical features, One-Hot Encoding was applied to maintain discrete class affiliation, while continuous features were normalized using z-score normalization. Due to the imbalance in y (as only about 11.2% of encounters are readmitted), we used said ratio for class weighting, which is supported natively by the scale_pos_weight parameter of XGBoost. The ratio of negative to positive samples were used as weights:

$$\mathrm{scale}\_\mathrm{pos}\_\mathrm{weight}=\frac{N_{\mathrm{neg}}}{N_{\mathrm{pos}}}=\frac{72326}{9086}\approx 7.96.$$

This strategy is able to alleviate the problem of under-sampling in the minority class but does not change the original data distribution.

**Figure 1:**
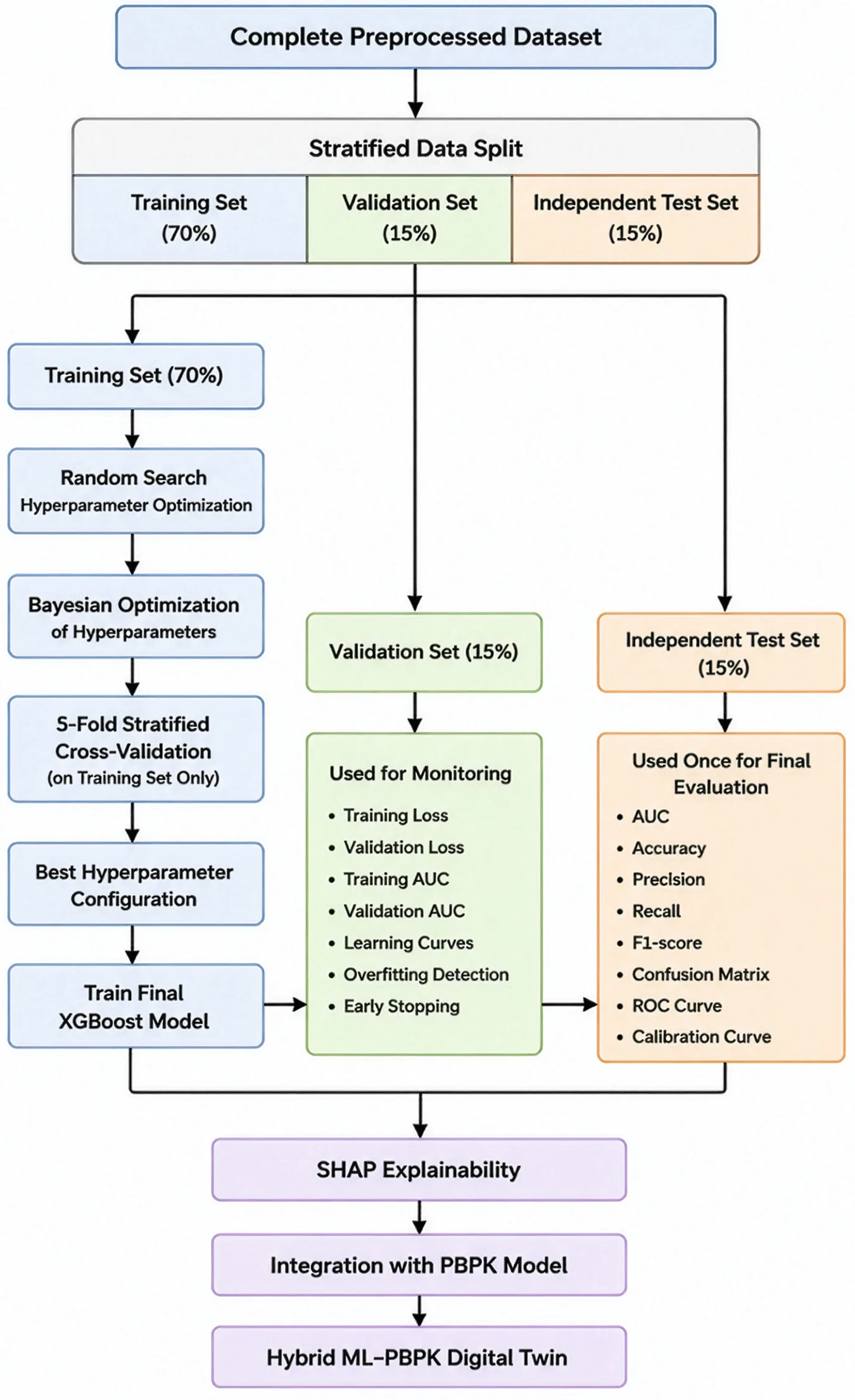
Machine learning model development and validation workflow, showing stratified data splitting, hyperparameter optimization, cross-validation, final testing, SHAP explainability, and integration with the hybrid ML–PBPK digital twin framework.

After preprocessing, the complete dataset was partitioned into training, validation, and independent test subsets, and the subsequent machine learning development pipeline adopted in this study is illustrated in Figure 1.

### 3.3 Feature engineering

Feature engineering concentrated on improving the representation of clinical and administrative data. Age groups were transformed to midpoints as a continuous approximation. Considering these, diagnostic codes were aggregated into clinically relevant groups to decrease sparsity and be related to standard disease classification. Changes in medications (dose escalation or reduction, initiation, discontinuation) were retained as binary markers of therapeutic change. Features regarding hospital- and ER-use (including the total number of inpatient visits, emergency department visits, procedures and lab tests) were included *because they are known to have relevance to readmission risk. The final feature matrix after encoding had more than 2,300 dimensions, enabling the model to learn complex associations across numerous clinical parameters.

For reproducibility, Table 2 highlights the main feature categories and their overall contribution to the final encoded feature space. A comprehensive list of input features, feature definitions, and encoding strategy is detailed in Appendix A (Table A1).

**Table 2:** Summarizes the primary feature categories and their relative contribution to the final encoded feature space.

Feature category	Examples	Approximate encoded features
Demographic Variables	Age, gender, race	∼50
Admission Characteristics	Admission type, source, discharge disposition	∼150
Hospital Utilization	Inpatient, outpatient, emergency visits	∼10
Diagnostic Categories	ICD-based diagnosis groups	∼500
Medication Variables	Insulin, metformin, medication changes	∼300
Procedures and Laboratory Utilization	Procedures, laboratory tests	∼20
Administrative Variables	Encounter-related information	∼20
One-Hot Encoded Categorical Variables	Expanded categorical representations	Remaining dimensions (>1,200)

### 3.4 Modeling development

Extreme Gradient Boosting (XGBoost) was selected as the primary predictive model because of its demonstrated effectiveness on high-dimensional tabular healthcare datasets, robustness to multicollinearity, and ability to model complex nonlinear interactions among heterogeneous clinical variables. Following data preprocessing and feature encoding, model development was performed using stratified sampling to preserve the original class distribution during model optimization and evaluation. Hyperparameter optimization was conducted during model development, while convergence behaviour was monitored using a dedicated validation procedure. The final predictive performance was subsequently assessed on an independent hold-out test set to provide an unbiased estimate of model generalization.

A stratified 70%/15%/15% partition was selected to provide sufficient data for model development while preserving independent validation and test sets for model monitoring and unbiased evaluation. Given the large cohort of 101,766 hospital encounters, this allocation ensured adequate representation of both outcome classes within each subset while maintaining strict separation between model optimization and final performance assessment.

**Figure 2:**
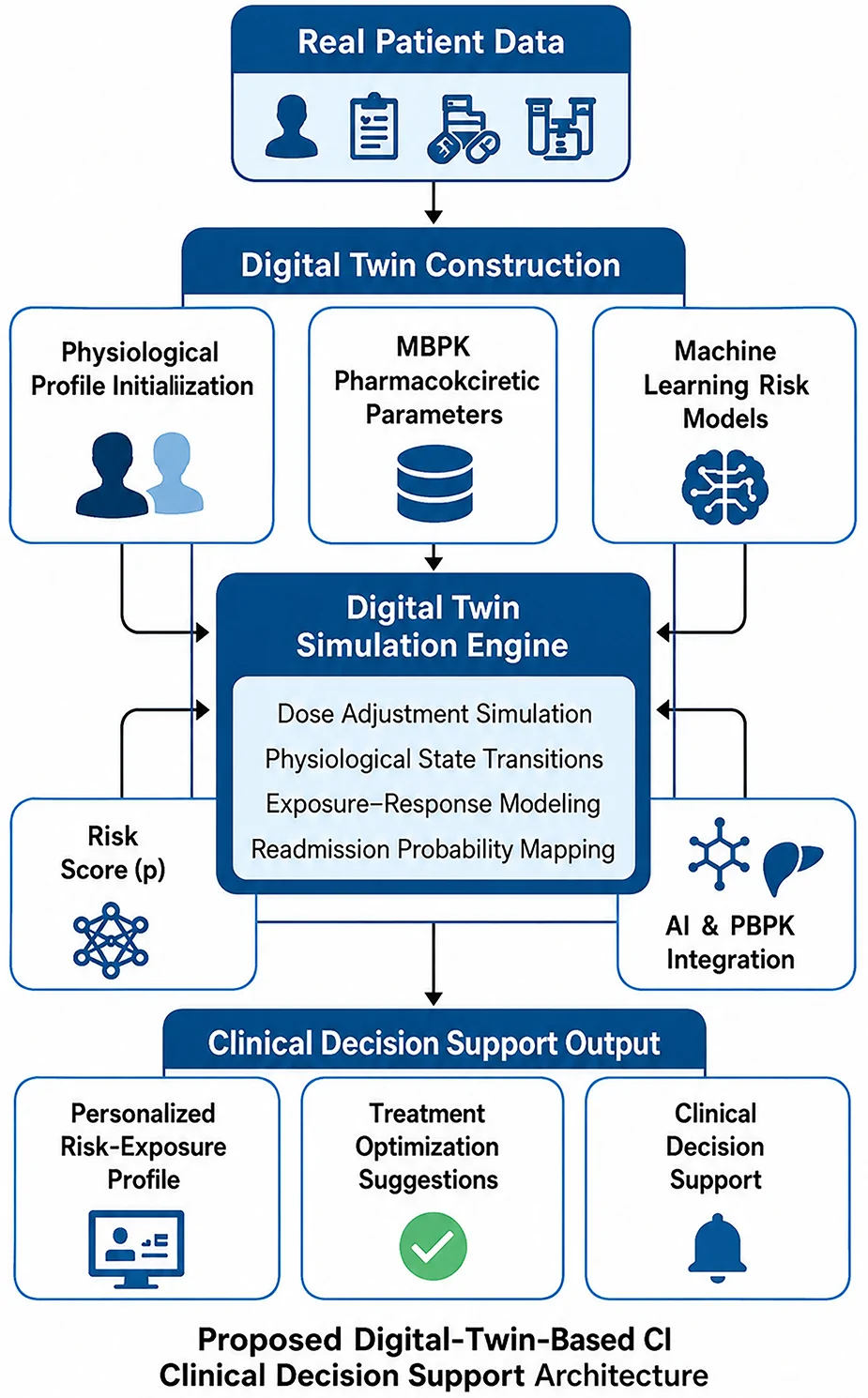
Proposed digital twin–based clinical decision support architecture illustrating the integration of real-world patient data, machine learning risk prediction, PBPK-driven exposure simulation, and personalized clinical decision outputs.

Beyond its widespread use in healthcare prediction studies, XGBoost was particularly suitable for the characteristics of the present dataset. Following categorical encoding, the feature space expanded to more than 2,300 variables, resulting in a sparse and high-dimensional tabular representation. XGBoost efficiently handles such feature spaces while capturing complex nonlinear relationships and naturally supporting cost-sensitive learning for imbalanced clinical datasets. Furthermore, its seamless integration with the TreeSHAP algorithm enables computationally efficient *post-hoc* interpretation of individual predictions, making it particularly suitable for the explainable Digital Twin framework proposed in this study (Figure 2).

To address the pronounced class imbalance, the scale_pos_weight parameter of XGBoost was determined according to the ratio between the majority and minority classes. Although the Synthetic Minority Oversampling Technique (SMOTE) was explored during preliminary experimentation, the final model employed cost-sensitive learning through scale_pos_weight because it preserved the original data distribution and avoided introducing synthetic observations that could potentially influence the clinical interpretation of model behaviour.

The final optimized XGBoost model employed 1,000 estimators, a maximum tree depth of 4, a learning rate of 0.05, a subsampling ratio of 0.8, a column sampling ratio (colsample_bytree) of 0.6, a minimum child weight of 25, a gamma value of 1.0, L1 regularization (reg_alpha) of 8.0, L2 regularization (reg_lambda) of 25.0, and the previously determined class-weight parameter (scale_pos_weight = 7.96). These hyperparameters were selected through the optimization strategy described in Section 3.15 to balance predictive performance, model complexity, and generalization while reducing the risk of overfitting. The model minimized the binary logistic loss function:

$$L=-\overset{n}{\underset{i=1}{\sum }}[y_{i}\log({\hat{\;p}}_{i})+(1-y_{i})\log(1-{\hat{\;p}}_{i})]$$

where $y_{i}$ denotes the observed class label and ${\hat{\;p}}_{i}$ represents the predicted probability.

During model optimization, both training and validation performance were monitored using binary logistic loss together with the corresponding Area Under the Receiver Operating Characteristic Curve (AUC). These learning curves were used to evaluate optimization stability, convergence behaviour, and potential overfitting throughout the boosting process. The corresponding training dynamics are presented and discussed in Section 4.2. All experiments were performed using a fixed random seed (42) to ensure reproducibility of data partitioning and model training.

### 3.5 Threshold optimization

Although default binary classification typically uses a threshold of 0.50, imbalanced clinical datasets often require customized thresholds to achieve clinically meaningful performance. A threshold sweep was performed across the interval. t∈[0.02,0.50] Increments of 0.01. For each threshold, predicted probabilities were transformed into class labels as follows: d into class labels as follows:

$${\hat{y}}_{t}=\{\begin{array}{cc} 1, & \hat{p}\geq t\\ 0, & \hat{p}<t \end{array}.$$

Performance metrics such as precision, recall, F1-score and accuracy were computed on the validation set. Trade-off between the sensitivity and specificity for threshold was optimal at t = 0.50 so, we did not adjust it. We achieved AUC of 0.686, recall of 0.60 and F1 = 0.2817 for naive model developed by us on the test set. All data preprocessing, resampling and hyper-parameter tuning were performed within folds so as not to spill information to the validation and test set. We did examine threshold optimization, where sensitivity specificity trade-offs were analyzed, but the default value of threshold t = 0.50 was chosen to enhance the interpretability of our results and facilitate model comparison.

### 3.6 Model explainability

Explainability was tested with SHAP (Shapley Additive explanations), a technique that dissects each prediction to show how much each feature moves it. For each example x SHAP writes the model output as:

$$f(x)=\;\emptyset_{0}+\overset{d}{\underset{i=1}{\sum }}\emptyset_{i},$$

where is the base line expectation and $\phi_{i}$ measures the contribution of the feature i. The most globally important predictors were identified using SHAP summary plots, and waterfall plots provided instance-level explanations. Important predictors were prior inpatient, discharge disposition, emergency visit and medication variables, which are also based on clinically known determinants of readmission.

### 3.7 PBPK model formulation

A physiologically based pharmacokinetic (PBPK) model was developed to estimate drug disposition in changing physiological environments. A one-compartment model with first-order absorption and elimination described by the equation:

$$\frac{dA_{\mathrm{gut}}}{\mathrm{dt}}=-k_{a}A_{\mathrm{gut}},\frac{dA_{\mathrm{plasma}}}{\mathrm{dt}}=k_{a}A_{\mathrm{gut}}-k_{e}A_{\mathrm{plasma}},$$

where $A_{\mathrm{gut}}$ and $A_{\mathrm{plasma}}$ Denote the drug amounts in the gastrointestinal tract and systemic circulation, respectively. Plasma concentration was computed as:

$$C(t)=\frac{A_{\mathrm{plasma}}(t)}{V_{d}}.$$

Simulations were performed in normal, renal impaired, hepatic impaired and low weight subjects, adjusting elimination or volume parameters. AUC was used to measure drug exposure levels:

$$\mathrm{AUC}=\overset{24}{\underset{0}{\int }}C(t)\;\mathrm{dt}.$$

Material differences in exposure were found throughout physiologic scenarios, suggesting the importance of accounting for pharmacokinetics within clinical prediction.

PBPK models were developed for metformin, which we put forth as a prototype first-line antidiabetic drug because of its wide clinical use and strong renal dependency. The PBPK component is intentionally simplified to serve as a relative exposure stratification mechanism rather than a dosing optimization tool. The goal is not pharmacometrics precision but physiological differentiation sufficient to contextualize machine-learning–derived readmission risk within a Digital Twin framework.

### 3.8 Digital twin integration

In this study, a Digital Twin is operationally defined as a patient-specific computational construct that integrates real-world clinical data with mechanistic simulation to enable individualized scenario analysis beyond static risk scoring. The Digital Twin combines ML-generated risk predictions and PBPK-based drug-exposure measurements to form a multi-dimensional description of an individual. For every encounter, the model outputs a predicted probability of readmission $\hat{\;p}$ and a corresponding set of personalized exposures is produced by the PBPK module. These are combined to build a risk–exposure phenotype, defined as:

$$D(x)=(\hat{\;p},\mathrm{AUC}),$$

and enabling clinicians to predict readmission by branching drug responses. This fusion provides a mechanistic explanation for predictions and promotes patient-specific treatment planning.

**Figure 3:**
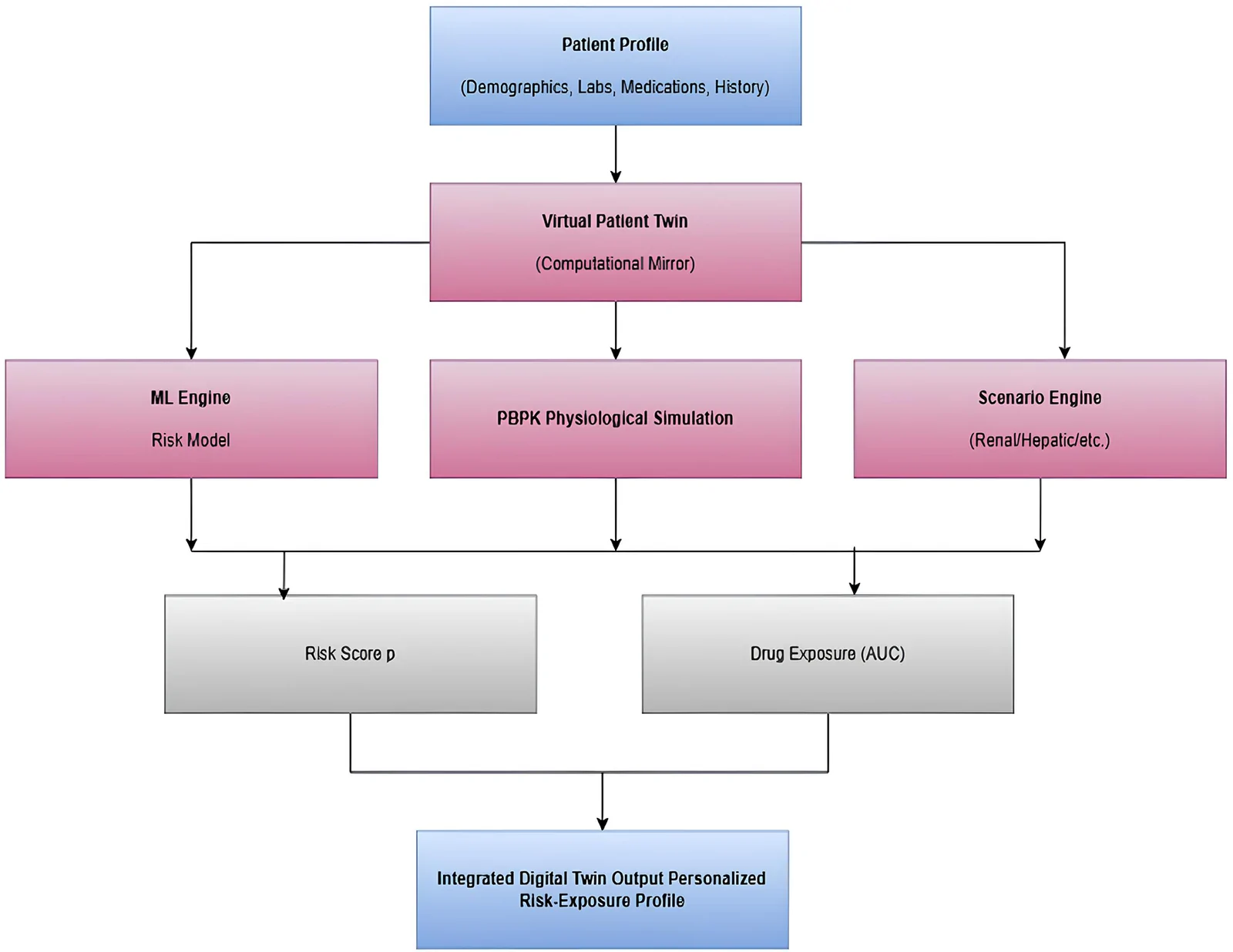
Conceptual digital twin workflow showing parallel machine learning–based risk modeling and PBPK-based physiological simulation converging into an integrated personalized risk–exposure phenotype.

This integration was purposefully done to take advantage of the synergistic relationship between machine-learning prediction and mechanistic pharmacokinetics. Rather than integrating the models at a feature level, the integration happens at the Digital Twin layer by creating a risk-exposure phenotype (Figure 3). The ML component handles identification of high-risk readmission patients from high dimensional data derived from EHR, and the PBPK component describes personalized drug exposure under a variety of physiological situations. This allows the framework to take into account the predictive and physiological aspects of the patient's status at once. With this configuration, the resulting Digital Twin can enable scenario-based analysis, physiological grounding of risk, and mechanistic clinical reasoning, all of which are major sources of synergy with the ML part and is a key advantage over purely predictive models.

The decision to integrate machine-learning and PBPK outputs at the Digital Twin layer rather than at the feature level was intentional. The present study was designed as a modular proof-of-concept framework in which predictive and mechanistic components remain independently interpretable and separately verifiable. This architecture allows machine-learning predictions and pharmacokinetic simulations to be evaluated using their respective validation strategies before integration into a unified patient representation. Furthermore, the underlying EHR dataset does not contain patient-specific pharmacokinetic measurements or longitudinal concentration profiles that would support rigorous calibration of tightly coupled pharmacokinetic learning models. Consequently, a modular integration strategy was considered the most appropriate design choice for the current study.

### 3.9 Workflow overview

In this work, we present an integrated workflow that combines data preprocessing, feature engineering, predictive modeling as well as mechanistic simulation and construction of a Digital Twin in a single analytical pipeline. Early pre-processing includes standardized imputation, encoding, and normalization techniques to maintain uniformity in data quality across all admissions. These operations help with noise reduction, attempt to remove some of the bias introduced by missing values and make it easier for the models to converge. Feature engineering creates a structured form of demographic, clinical, and medicine-based predictors, resulting in a high-dimensional input matrix as an input of gradient boosting tree modeling. Model development.

Stratified splitting of the dataset is used to avoid overfitting while tuning hyperparameters and selecting a model. The what ⇒ Parameters you want to keep track of generalization performance on iterations (threshold optimization or monitoring weigh on the threshold) steps.

Simultaneously with the ML workflow, PBPK simulations are run to perform in silico personalizations of drug concentration–time profiles and exposure metrics across different physiological environments. These simulations are not reliant on EHR-based predictors and also cover the spread of pharmacokinetics that is not represented in administrative hospital datasets. These outputs are combined at the Digital Twin level to provide risk–exposure phenotypes for each patient, calculated as the predicted readmission probabilities and the AUC generated using PBPK modelling. The integration point is designed to provide interpretable components and a physiological customization.

We did all data manipulation in Python (v 3.10) using commonly used scientific libraries to facilitate replicability. We designed the pipeline as a modular framework with separated preprocessing, model and simulation stages to scale the number of datasets or physiological scenarios we would be able to process. This framework is easy to work with and supplies a solid reproducible basis for hybrid predictive modeling in diabetes care.

### 3.10 Model validation strategy

After splitting the data into the training (70%), validation (15%) and independent test (15%) partitions, a five-fold stratified cross-validation scheme was applied only to the 70% training portion to demonstrate the strength and stability of the optimized XGBoost model. This was implemented such that each partition served once as an internal validation partition for the model in question, while all the other partitions served to train it. Note that the validation subset (15%) was used only to perform hyperparameter tuning, and for training-stop, whereas the test subset (15%) remained untouched and was employed only at the very last step for the model performance test.

### 3.11 Model evaluation metrics

The model's performance was evaluated by means of a group of metrics that is standard practice in the clinical prediction literature. Accuracy, precision, recall and F1-score were computed to analyze different components of the classification performance. We emphasized recall since clinical practice dictates that minimizing the number of false negatives (missed high risk patients) is paramount, especially in a readmission prediction scenario. We measured the discrimination of the model in classifying readmitted vs. non-readmitted patients at different thresholds of probability by means of the receiver operating characteristic curve (ROC) and area under the ROC curve (AUC). Calibration was analyzed through reliability diagrams, which compare model predicted probabilities against observed event rates through a line of agreement. This combination of evaluation measures gives a comprehensive summary of the discrimination, calibration and the clinical utility of the model, allowing for a clear assessment of its potential application in clinical practice.

### 3.12 Software and computational environment

All analyses were performed using Python (version 3.10) with a controlled conda environment to ensure reproducibility. Primary libraries were NumPy (v1.24), pandas (v1.5.x), scikit-learn (v1.3.x), XGBoost (v2.0.x), SHAP, and Matplotlib. PBPK simulations were performed with in-house developed Python modules on normal pharmacokinetic equations. The model training and evaluation were run on a workstation with an Intel i5 processor and 16 GB RAM, which was adequate to process the dataset and simulation loads. To obtain deterministic results, random seeds were set to be the same on preprocessing, training, and evaluation of the model. The workflow was modularized to accommodate replication, and all code scripts were organized for transparency and replicability. We used a versioning system based on Git to record changes and ensure experimental consistency during development.

### 3.13 Ethical considerations and data governance

This work used the de-identified publicly available diabetes readmission database that conforms toHIPAA regulations and US privacy laws. No patient identifying information was available (names, locations, contact information). Because the data is released with strict access controls and no potential for re-identification, no individual patient could be identified. Data analysis and all modeling and simulation were performed at the population/aggregate level using secure local resources with no data transfer to external servers. Because of the nature of the clinic database for secondary use and the lack of any human interaction, institutional review board approval was not needed. However, to ensure compliance with minimization and confidentiality rules, only those variables required to build the model were used. This approach was constructed to promote transparency, reproducibility, and fairness and to reduce risks of bias from factors inherent in clinical data sets.

### 3.14 Handling class imbalance and justification of resampling strategy

In the diabetes readmission dataset, the ratio between non-readmitted and 30-day readmitted classes is extremely imbalanced (about 89.4% vs.10.6%). Without addressing this imbalance, accuracy increases at the expense of recall that is usually unacceptably reduced in clinical meaningfulness. To reduce these consequences, we employed a class imbalance mitigation strategy approach to the study. First, the scale_pos_weight parameter of XGBoost was tuned as the inverse of prevalence ratio in order to balance the gradient updates during model building. During initial modeling and cross-validation experiments, Synthetic Minority Over-sampling Technique (SMOTE) was also investigated. However, in the final reported model, we used only the cost-sensitive learning of XGBoost, using the scaleposweight parameter, in order to retain the original distribution of data and prevent the creation of some potential artifacts when synthetic samples are created. SMOTE was examined in the course of exploratory experiments within cross-validation folds, which we did, but in the end, our reported model mainly used class weighting (scale_pos_weight) to avoid synthetic data leakage.

### 3.15 Hyperparameter optimization and model selection strategy

Rigorous hyperparameter optimization was implemented to facilitate fair model comparisons and mitigate the potential for overfitting. The model development strategy followed a strict separation between model optimization and final performance evaluation. Hyperparameter tuning was performed exclusively during model development, while the independent test set was reserved solely for the final assessment of predictive performance. This strategy ensured that no information from the hold-out test set influenced model selection or parameter optimization.

The proposed XGBoost model was optimized in two sequential stages. Initially, a randomized search was conducted over a broad hyperparameter space comprising the learning rate, maximum tree depth, number of estimators, subsample ratio, column subsampling ratio (colsample_bytree), minimum child weight, gamma, L1 regularization (reg_alpha), and L2 regularization (reg_lambda). Subsequently, Bayesian optimization was employed to refine the most promising hyperparameter combinations using the Area Under the Receiver Operating Characteristic Curve (AUC) as the primary optimization objective while maintaining recall as a secondary clinical constraint to preserve sensitivity for identifying high-risk patients.

Following hyperparameter optimization, the final XGBoost model was trained using the optimized configuration (max_depth = 4, learning_rate = 0.05, subsample = 0.8, colsample_bytree = 0.6, min_child_weight = 25, gamma = 1.0, reg_alpha = 8.0, reg_lambda = 25.0, scale_pos_weight = 7.96). During training, validation-based early stopping with a patience of 20 boosting rounds was incorporated to automatically terminate optimization once the validation AUC no longer improved, thereby reducing unnecessary model complexity and mitigating the risk of overfitting.

To evaluate model robustness, five-fold stratified cross-validation was performed exclusively on the 70% training subset after the initial 70%/15%/15% stratified data split. The independent validation subset was reserved for hyperparameter optimization and early stopping, while the independent test subset remained completely unseen throughout model development and was used only once for the final performance evaluation. This validation strategy maintained strict separation between model optimization and performance estimation while providing complementary evidence of model robustness across different training partitions.

For comparison, the baseline Logistic Regression and Random Forest models were optimized using grid search with five-fold stratified cross-validation over their respective hyperparameter spaces. To ensure full reproducibility, all experiments were conducted using a fixed random seed of 42.

### 3.16 Cross validation, generalizability assessment and overfitting control

The model's stability and robustness was further evaluated using a 5-fold stratified cross-validation process on only the 70% training data, after the data was partitioned as stratified train (70%), validate (15%) and independent test (15%) sets. Stratification ensures that all classes represented in the dataset are similarly represented in each fold, while the validate set and the independent test set were not used at all during cross validation. The validate set was used only to tune the hyper parameters of the model and for model's early stopping and the independent test set was only used once for performance assessment. Fold-wise AUC along with their means and standard deviations were also used for model robustness.

Early stopping with multiple regularization techniques: maxdepth, minchildweight, gamma, regalpha, reglambda, subsample, colsamplebytree were all employed in order to minimize the risk of over-fitting.

Binary logistic loss andAUC values of training and validate set were computed through the process of model training. When validation performance stopped improving training was stopped auto. Learning curves were analyzed in order to show stable optimization process, controlled model complexity, and consistence between training and validate performance.

### 3.17 Statistical significance testing and confidence interval estimation

We computed 95% confidence intervals (CI) for AUC, precision, recall and F1-score values with non-parametric bootstrapping to test whether the improvements of the XGBoost model over baseline models were statistically significant. Pairwise comparisons between models were performed by the DeLong test to assess the statistical significance of correlated ROC curves. The null hypothesis tested whether there was no difference in predictive performances between models. An AP-value < 0.05 was deemed statistically significant. Incorporating significance tests additionally validates that performance differences are statistically attributable to methodological improvements as opposed to sample variability. This rigor is aligned with and supported by methodological standards in CBM.

### 3.18 PBPK model verification and parameter sensitivity assessment

The PBPK models were validated with available pharmacokinetic parameters in the literature for metformin exposure under varying physiological scenarios. Pharmacokinetic parameters—renal clearance, hepatic extraction ratio, tissue partition coefficients and absorption rate constants—were tested against known reference values to verify biological realism. Model calibrations were achieved by modifying partition coefficients in a range of physiological values to reproduce observed baseline AUC within reported clinical data. Sensitivity analysis was also performed by varying the following parameters (ka, ke, CLr) and ±20% to determine their effect on exposure metrics, e.g., area under the curve (AUC), maximum concentration (Cmax), time-to-peak (Tmax). These analyses validated that renal clearance was the predominant driver of drug exposure, and therefore it was selected as a mechanistic input for downstream digital twin risk scoring. This validation confirms that those features of the PBPK used in the Integrated ML–PBPK Framework are physiologically meaningful and not numerically driven.

### 3.19 Explainability framework using SHAP for clinical interpretability

Model interpretability was maintained with SHAP (Shapley Additive exPlanations) that explains changes in predicted risk of readmission by features of a single patient on game theoretical grounds. Beta values for global and individual-level prediction drivers were plotted using SHAP summary plots, dependence plots and visualized as a waterfall. Characteristics including previous inpatient visits, emergency department visits, disposition at discharge, and insulin/metformin treatment, emerged as significant predictors aligning with clinical intuition. SHAP interaction values were employed to investigate nonlinear effects and possible synergy of physiological factors and medication status. This interpretability framework enhances clinician confidence and enables potential downstream incorporation into decision-support pipelines.

The two alternative forms of interpretability within the proposed approach must be identified and contrasted. While SHAP offers a *post-hoc* model explanation of how each of the EHR-derived input variables contribute to the prediction of patient readmission risk via machine learning, the PBPK approach offers mechanistic physiological interpretability of the biological processes driving drug exposure given varying physiological states. These two alternative interpretations answer separate but complementary questions: SHAP explains why an individual is predicted to be at high risk, while PBPK explain why physiological variation may drive an individual treatment exposure which may contribute to this risk. Integrating both answers provides a singular risk-exposure explanation which could not be gained from either method individually, integrating both data driven and mechanistic interpretations within the Digital Twin, and giving deeper understanding towards the support of clinical decision making on an individual level.

### 3.20 Proposed digital twin architecture

The novel framework combines machine learning founded clinical prediction and physiology-based pharmacokinetic simulation into a singular DT structure describing personalized readmission risk with variability in drug exposure.

The architecture includes four main modules: (i), a data preprocessing and feature engineering module that preprocesses structured clinical inputs, (ii) a machine learning prediction module of the probability of 30-day readmission, (iii) A PBPK simulation module to generate drug concentration time profiles under varying patient physiological state, and (iv) an integration layer which combines results from both models.

Before they reach the model input, raw patient data are preprocessed by imputation, encoding, scaling, and feature extraction operations to yield a high-dimensional matrix for training.

The ML model analyzes this matrix to provide a continuous risk score; meanwhile, the PBPK module calculates personalized exposure metrics (e.g., AUC) by modifying model parameters according to renal, hepatic or weight-related disabilities. These parts are pooled by the Digital Twin integration layer to produce two-dimensional phenotype $D(x)=(\hat{\;p},\mathrm{AUC})$ for each of the patients. This architecture is designed to support a physiology-based interpretation of the predicted risk and serves as a groundwork for a personalized decision-support system, free from known scenario testing and simulation-driven treatment planning.

## 4 Results

### 4.1 Dataset characteristics and cohort description

**Figure 4:**
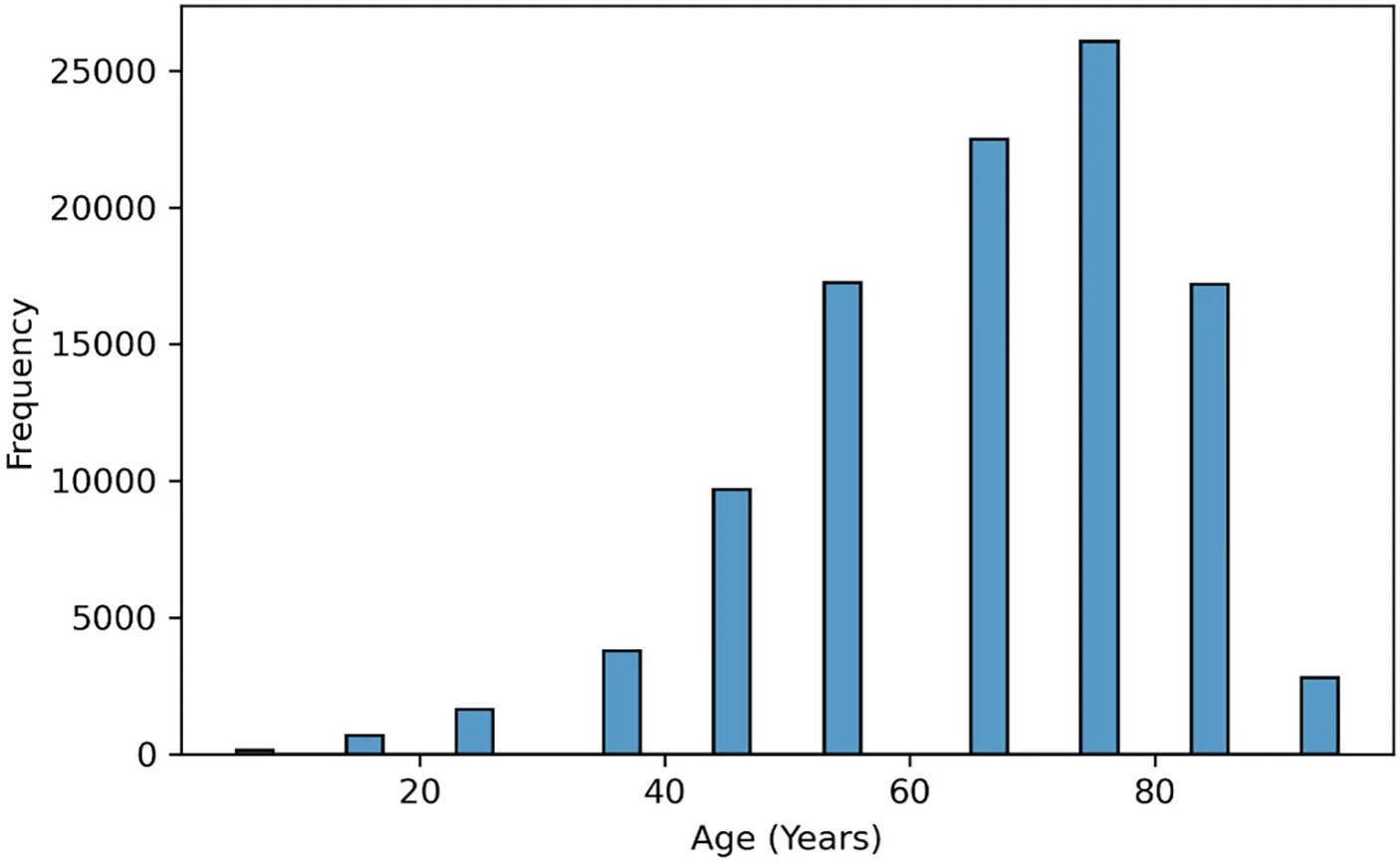
Age distribution of the inpatient encounters highlights a predominance of older adults, reflecting the typical demographic profile of hospitalized type 2 diabetes patients.

**Figure 5:**
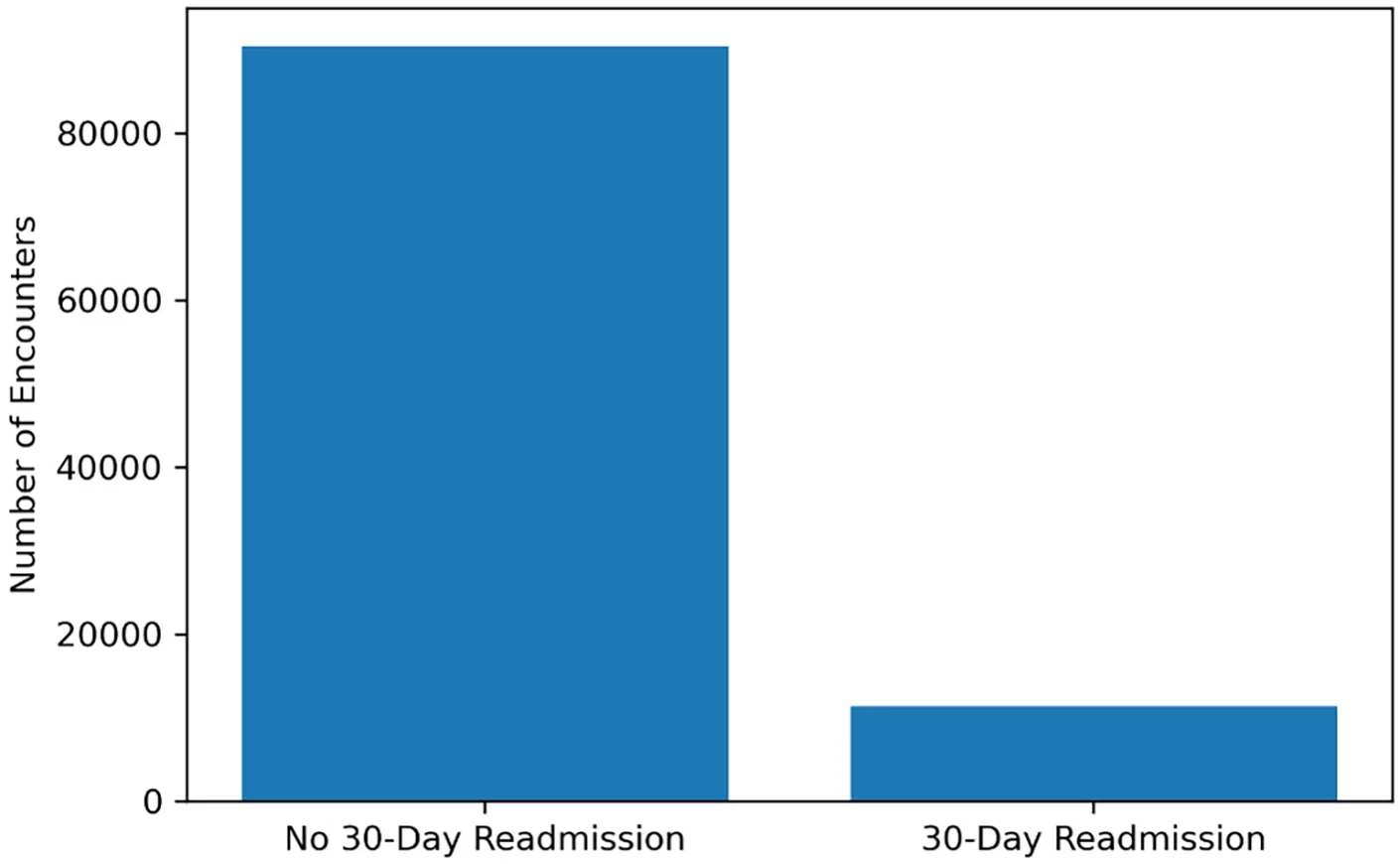
Class distribution showing an imbalance between readmitted and non-readmitted cases.

**Figure 6:**
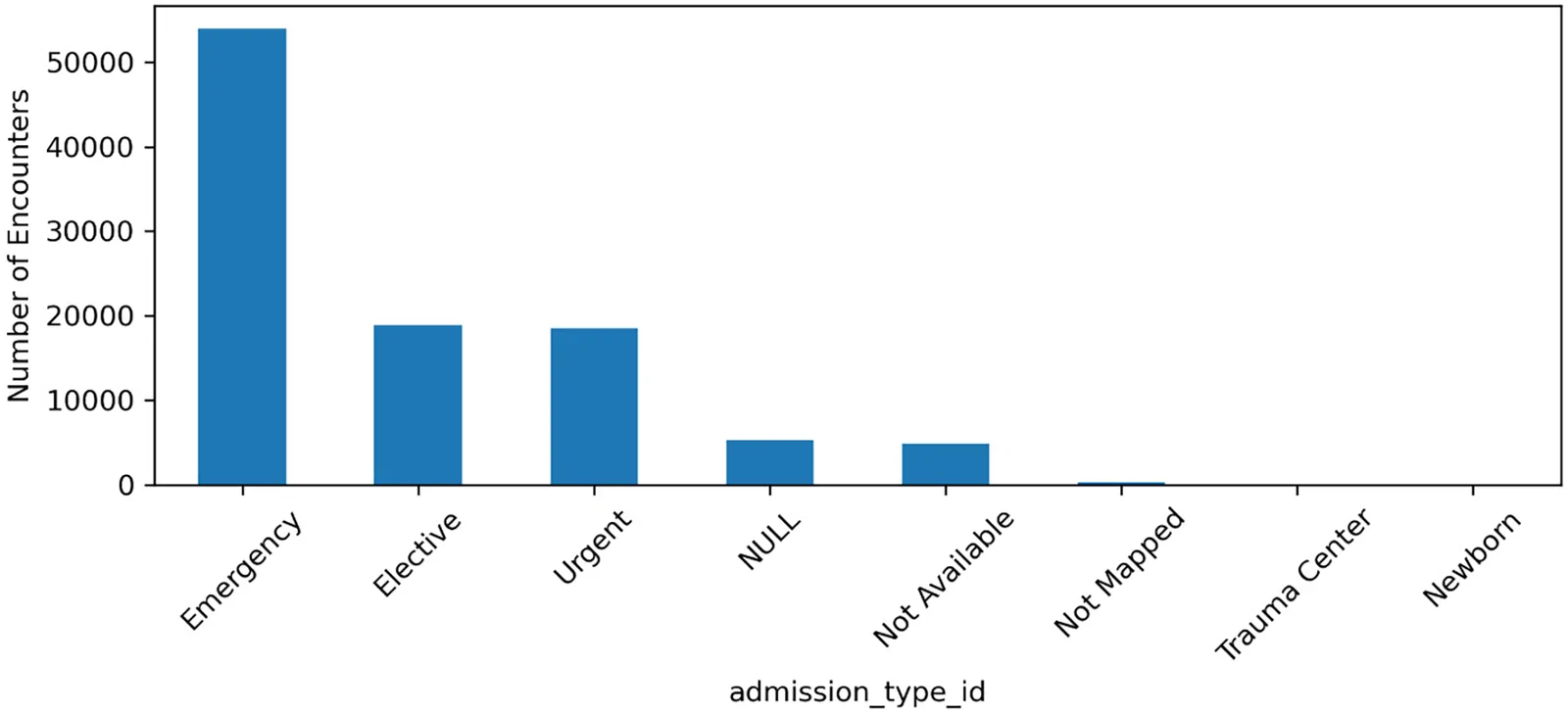
Distribution of admission types, emergency admissions dominate the cohort, indicating acute clinical presentations as a major driver of hospital encounters in diabetes patients.

**Figure 7:**
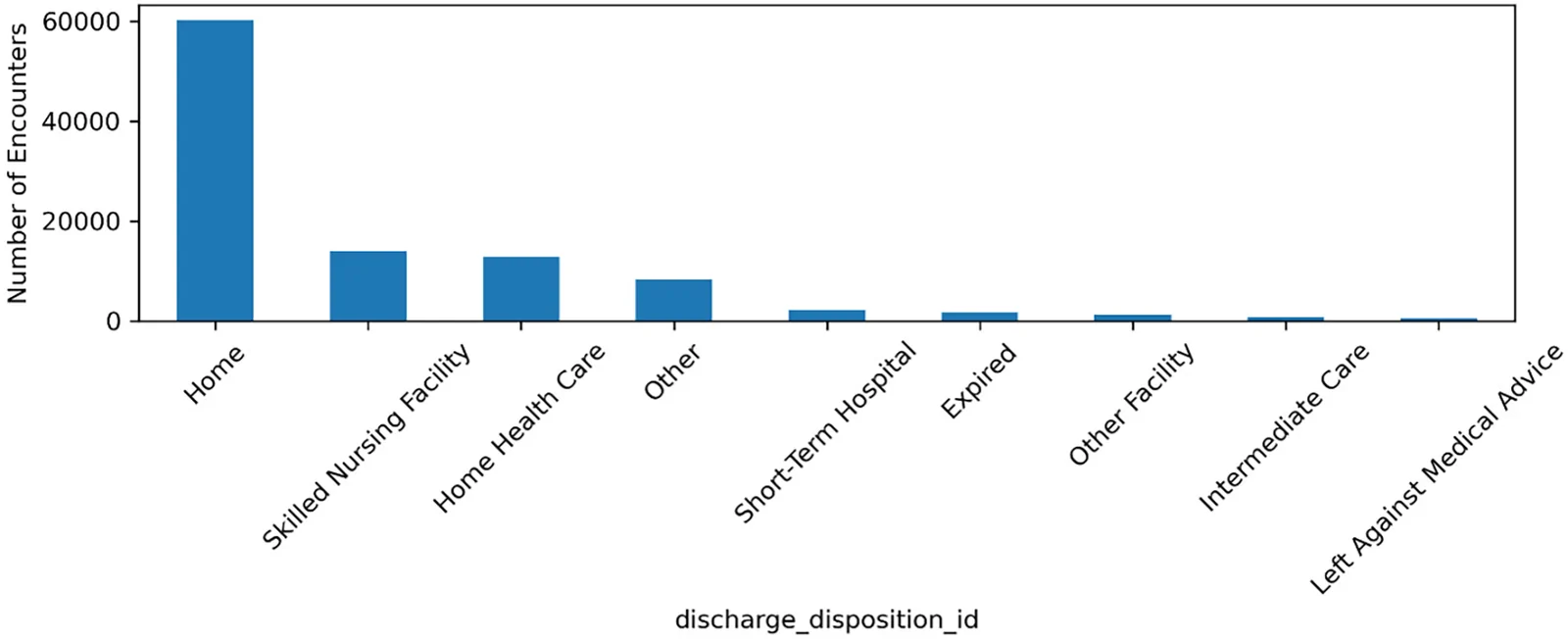
Distribution of discharge disposition, patterns reveal variability in post-hospital care pathways, which are key determinants of readmission risk.

**Figure 8:**
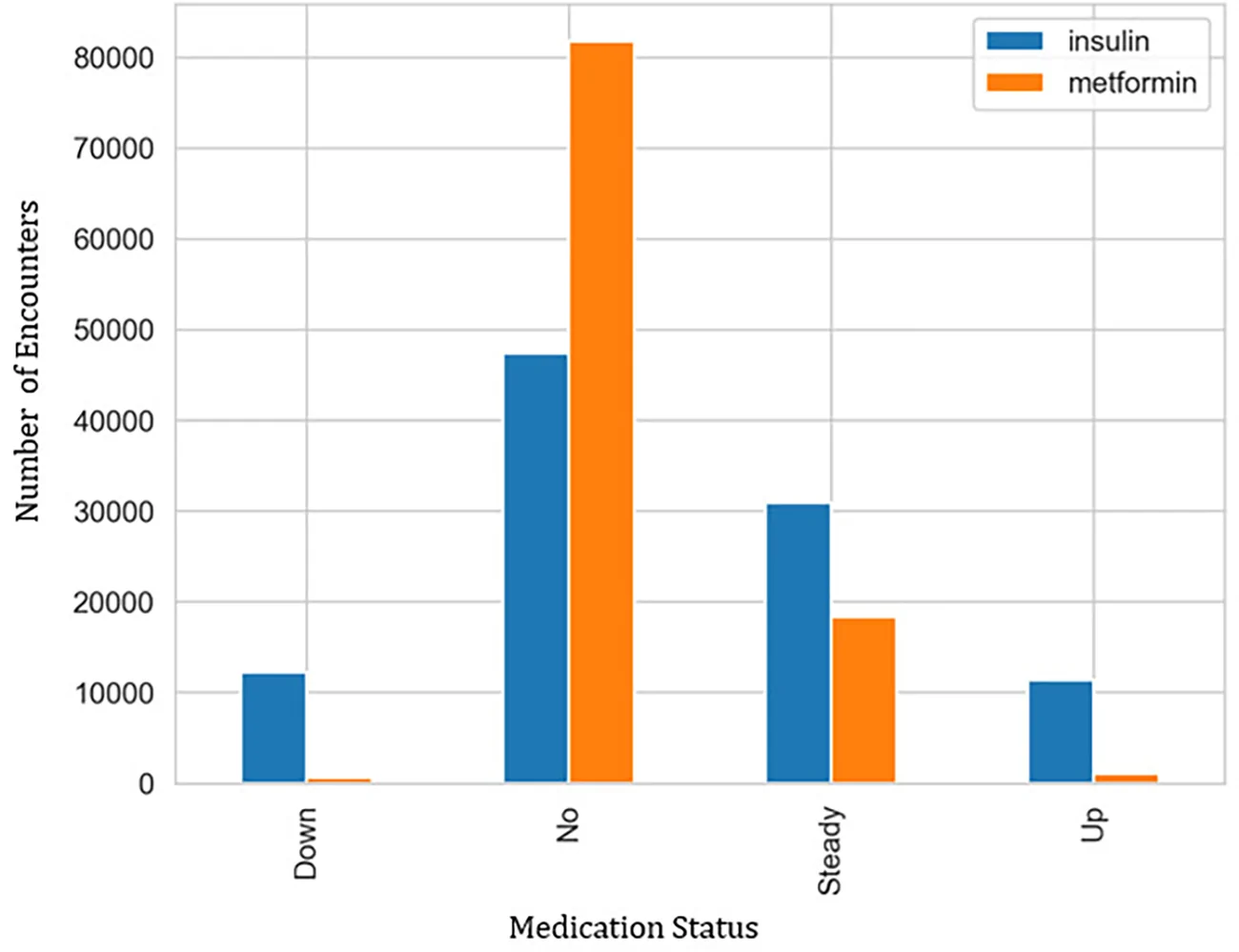
Distribution of insulin and metformin medication status. Medication adjustments during hospitalization are frequent and serve as indicators of disease instability and therapeutic response.

The analytic cohort was generated from the Diabetes 130-US Hospitals data after excluding records with missing essential identifiers or questionable discharge destination and included around 81,000 hospital admissions. Figures 4–8 show the demographic and utilization variables. (AgeDistr, ClassDistr, RACE class, DPTYPED, Disch disp, Insulin&DMET Meds). This population was balanced with respect to sex, and the range of ages encountered for the adult Type-2 population was normal with majority of encounters in the 50–80 range. There was quite a large variation among inpatient utilization metrics: number of previous inpatient admissions and A&E attendances varied considerably with a small proportion of people occupying a significant proportion of these resources (see utilisation distributions). The main outcome variable, the proportion of all-cause unplanned 30-day readmission, was present in 11.2% of the cases and there was therefore a class imbalance that was taken into account during modelling. These descriptive analyses indicate that the data is indeed rich in terms of complicated, multimorbid inpatient diabetes care and supports the idea of building capacity predicting models that can simulate a mechanism.

Demographic of the study cohort; Histogram of age (using midrange calculation) for 101,766 inpatient visits among those with a diabetes diagnosis; A higher number of elderly individuals is evident, but this is likely representative of the current nature of Type 2 diabetes and risk of hospitalization.

Frequencies of admission by emergency, urgent and elective routes are presented in the figure. Most of these are emergency admissions, predominantly representing acutely decompensation symptomatic patterns, again characteristic of diabetes.

Bar chart showing 5 min averages of Insulin (Right) and Metformin (Left)—Stratiﬁed as no change, up, or down of therapy. These medication changes are common throughout the index admission and are due to a mix of poor glycemic control and changes penciled in based on clinical status. These therapeutic changes are clinically important indices of disease severity and have been found to be related to an increased likelihood of readmission post-discharge, particularly among patients with underlying renal or metabolic compromise.

### 4.2 Model training and learning dynamics

**Figure 9:**
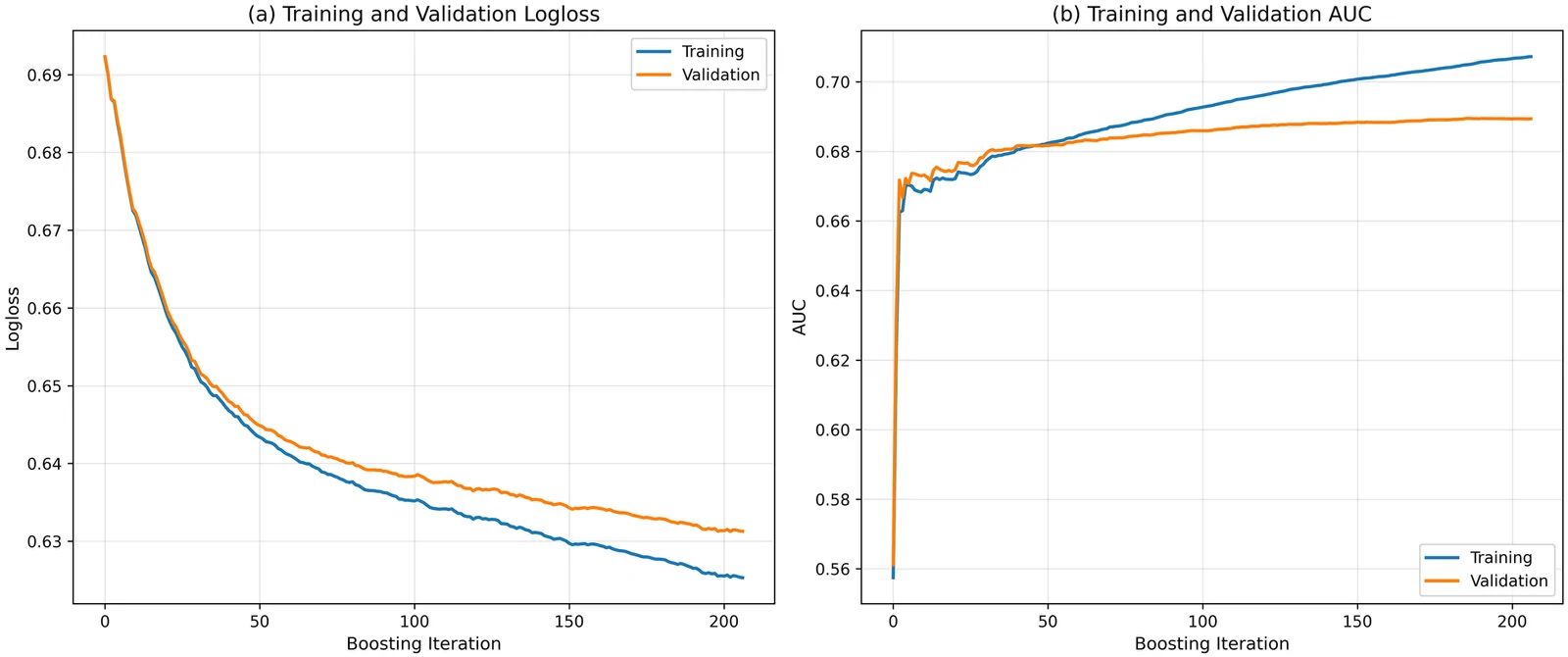
Training dynamics of the optimized XGBoost model. **(a)** Training and validation binary logistic loss across boosting iterations showing continuous reduction in loss during model optimization. **(b)** Training and validation AUC across boosting iterations. The validation AUC improves rapidly during the initial boosting iterations and reaches a plateau after approximately 50 iterations, whereas the training AUC continues to increase gradually until early stopping is triggered. This divergence indicates residual overfitting despite the application of regularization and validation-based early stopping, suggesting that additional boosting iterations primarily improved the model fit to the training data without corresponding gains in validation performance.

Predictive models were trained using the optimized hyperparameters identified through the model selection procedure described in Section 3.14. During model development, binary logistic loss and the Area Under the Receiver Operating Characteristic Curve (AUC) were continuously monitored on both the training and validation subsets to evaluate optimization behaviour and model generalization. To limit excessive model complexity, validation-based early stopping was incorporated, allowing training to terminate automatically once validation performance no longer improved. The resulting learning curves of the optimized XGBoost model are presented in Figure 9.

As shown in Figure 9(a), both the training and validation binary logistic loss decreased steadily throughout the boosting process, indicating effective optimization of the objective function. The validation loss closely followed the training loss, although a modest separation between the two curves became apparent during the later boosting iterations. Figure 9(b) demonstrates that the validation AUC improved rapidly during the initial boosting iterations and reached a plateau after approximately 50 iterations, whereas the training AUC continued to increase gradually until training was terminated by validation-based early stopping at 186 boosting iterations. This divergence between the training and validation AUC curves indicates the presence of residual overfitting, suggesting that the model established its generalization capability relatively early during training and that subsequent boosting iterations primarily improved the fit to the training data without producing corresponding gains in validation performance.

**Figure 10:**
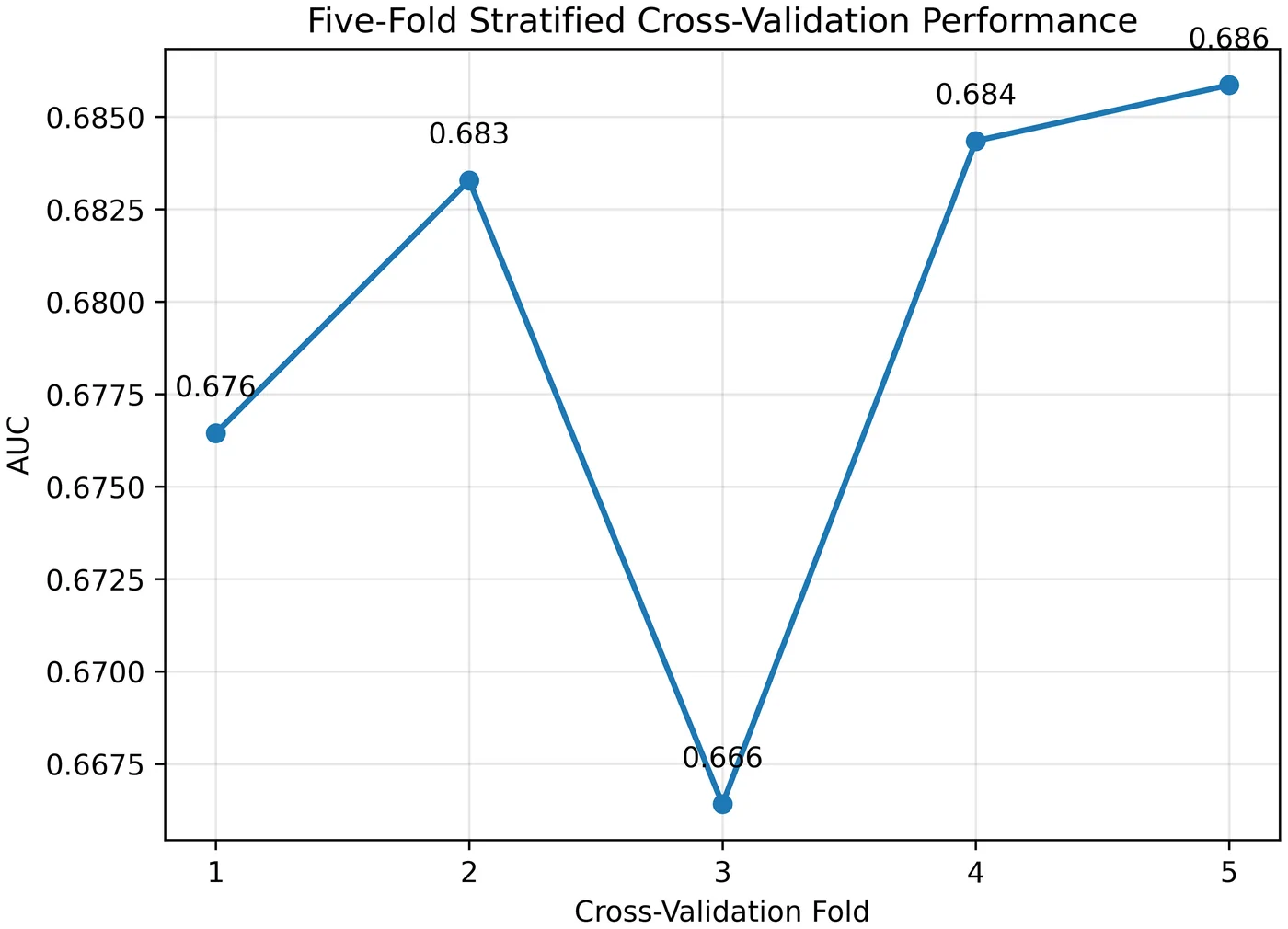
Stability of model performance across five-fold stratified cross-validation. Five-fold stratified cross-validation was performed exclusively on the 70% training subset during model development. Fold-wise AUC values demonstrate consistent predictive performance across different training partitions (mean AUC = 0.6793 ± 0.0072), indicating good model robustness and stability.

The final optimized XGBoost model employed the hyperparameter configuration identified during the optimization process, consisting of max_depth = 4, learning_rate = 0.05, subsample = 0.8, colsample_bytree = 0.6, min_child_weight = 25, gamma = 1.0, reg_alpha = 8.0, reg_lambda = 25.0, together with scale_pos_weight = 7.96 to address class imbalance. Validation-based early stopping selected the optimal model at 186 boosting iterations, corresponding to the highest validation AUC (0.6895). Although the learning curves indicate residual overfitting, the combined use of regularization, cost-sensitive learning, and validation-based early stopping limited further divergence between the training and validation performance. Furthermore, the optimized model demonstrated consistent performance across five-fold stratified cross-validation, achieving a mean AUC of 0.6793 ± 0.0072 (Figure 10), while maintaining comparable discrimination on the independent test set (Section 4.3). Therefore, the finalized model was considered sufficiently robust for subsequent SHAP interpretation, PBPK simulation, and Digital Twin phenotyping, while acknowledging the observed training dynamics as a limitation discussed in Section 5.

### 4.3 Predictive performance on the independent test set

The final optimized XGBoost model was evaluated on the independent 15% hold-out test subset, which remained completely unseen throughout model development, hyperparameter optimization, and model selection. As summarized in Table 3, the model achieved an Area Under the Receiver Operating Characteristic Curve (AUC) of 0.686, demonstrating moderate discriminative performance for identifying patients at risk of 30-day hospital readmission. This level of performance is consistent with previously reported machine learning models developed using structured electronic health record data for hospital readmission prediction.

**Table 3:** Baseline model comparison metrics.

Model	AUC	Recall	Precision	F1-score	Accuracy
Logistic Regression	0.63	0.45	0.17	0.22	0.86
Random Forest	0.65	0.51	0.19	0.25	0.87
**XGBoost (Proposed)**	**0**.**686**	**0**.**60**	**0**.**18**	**0**.**282**	**0**.**66**

Bold values indicate the performance metrics of the proposed XGBoost model.

The optimized model achieved a recall of 0.60, precision of 0.18, and an F1-score of 0.282, as reported in Table 3. Although the precision remained modest because of the highly imbalanced class distribution, the relatively higher recall indicates that the proposed model successfully identified a substantial proportion of patients at risk of readmission. From a clinical decision-support perspective, prioritizing recall is desirable because missed high-risk patients (false negatives) may have greater clinical consequences than additional false-positive alerts. The Precision–Recall curve further complements the ROC analysis by demonstrating the model's capability to identify the minority readmission class under severe class imbalance.

Calibration analysis (Figure 12) demonstrated generally good agreement between predicted probabilities and observed event frequencies across most probability intervals. Mild overestimation of risk was observed within selected intermediate probability ranges, where predicted probabilities were slightly higher than the corresponding observed event frequencies. The quantitative calibration measures presented in Table 3, together with the reliability diagram shown in Figure 12, indicate satisfactory agreement between predicted probabilities and observed outcomes, supporting the suitability of the optimized model for subsequent SHAP-based interpretation, PBPK integration, and Digital Twin–based clinical risk stratification.

“The proposed XGBoost model demonstrates improved sensitivity compared to baseline models, which is critical in clinical screening scenarios where minimizing missed high-risk patients is prioritized.”

Comparison of confusion matrices, showing the classification results. Sensitivity increased at normalized values after optimized value of threshold determination.

**Figure 11:**
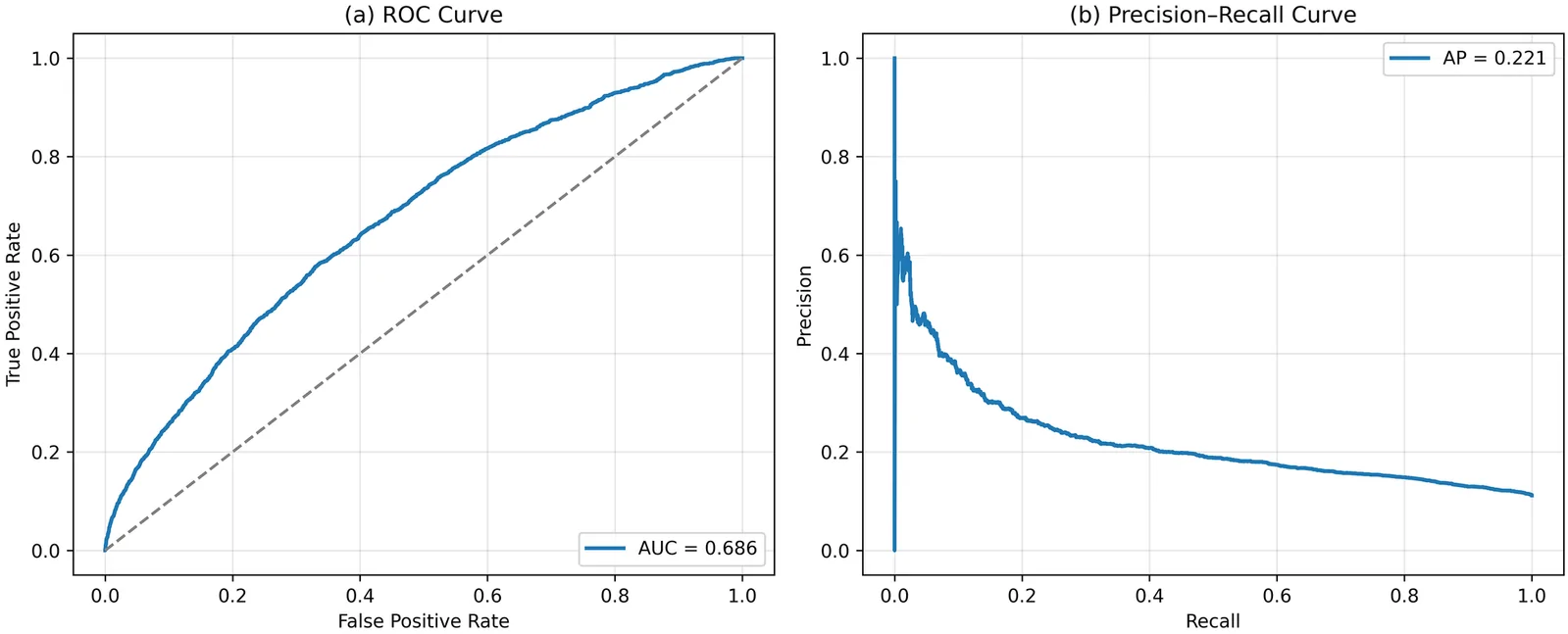
Predictive performance of the optimized XGBoost model on the independent test set. **(a)** Receiver Operating Characteristic (ROC) curve demonstrating an AUC of 0.686. **(b)** Precision–Recall (PR) curve illustrating classifier performance on the minority readmission class with an Average Precision (AP) of 0.221, providing complementary evaluation under class imbalance.

Performance of these measures is also described visually by confusion matrices as shown in Figure 13. The threshold for classification is biased towards greater sensitivity, identifying more of the true readmissions at the cost of less precision, reflecting the model's target screened application to find high-risk patients while accepting a proportion of false positive classification (Figure 11).

### 4.4 Comparative evaluation vs. baseline models

Comparison with baseline models demonstrates that the proposed approach achieves competitive performance. The re-trained logistic regression model achieved an AUC of approximately 0.63, while the random forest model achieved an AUC of approximately 0.65. In comparison, the proposed XGBoost model achieved an AUC of 0.68 (Figure 14 and Table 3), indicating improved discriminative capability. This performance places the model at the higher end of the commonly reported range for diabetes readmission prediction, while maintaining clinically meaningful sensitivity for identifying high-risk patients.

**Figure 12:**
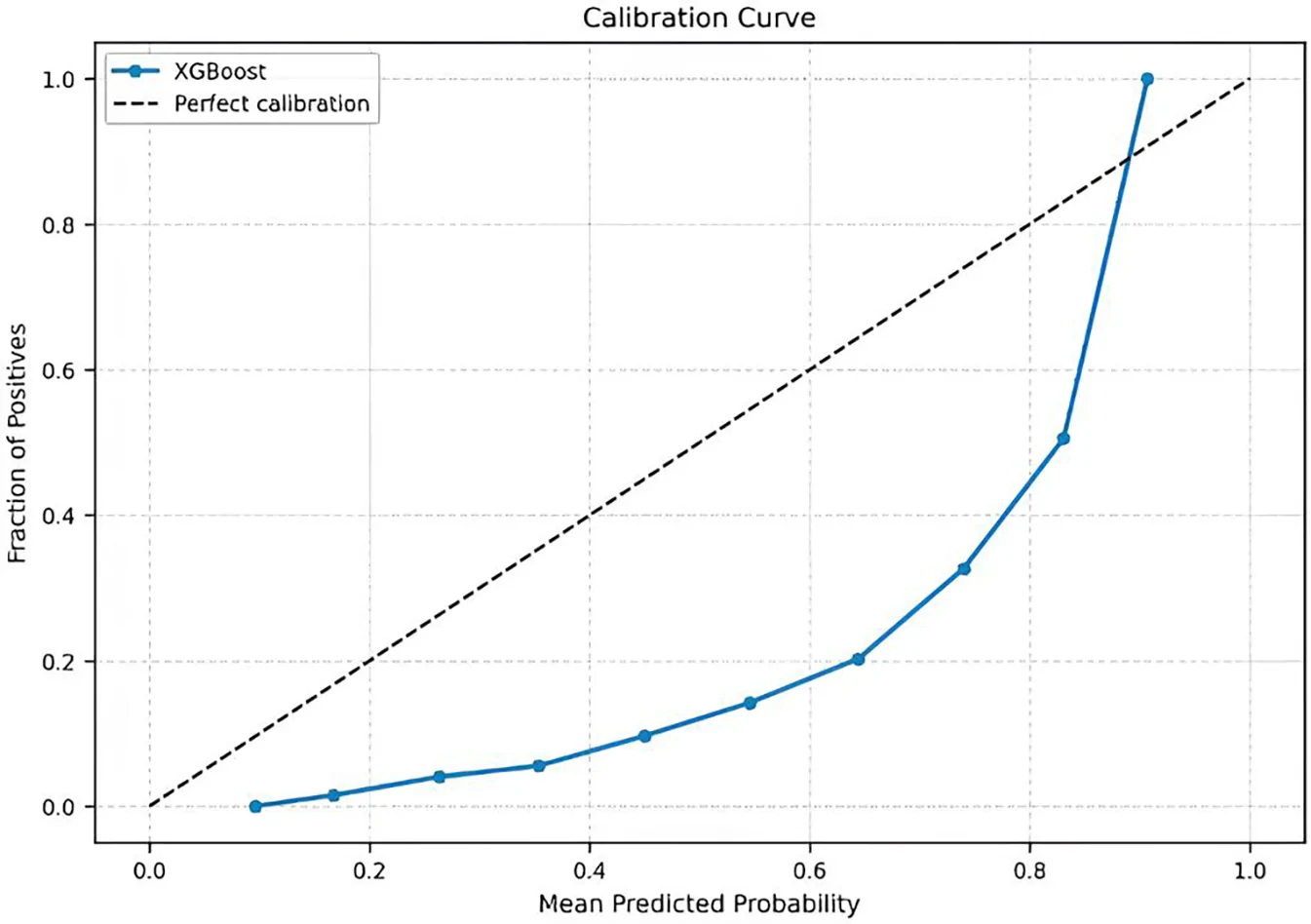
Calibration curve.

Absolute gains were largest in AUC and recall, with XGBoost achieving clinically meaningful increased sensitivity over logistic. The combined comparison graph (Figure 12) illustrates AUC, recall, and F1-score comparisons across models; the corresponding performance metrics are reported in Tables 3 and 4, while statistical significance testing is summarized in Table 5. Model comparison the more complex XGBoost model showed better performance than logistic regression, and pairwise comparisons of the models were reported in Table 5 (the nested CV and bootstrap methods are described in Section 3.16), which verified that the improvement on AUC by XGBoost was across the resampling process, and unlikely to be due to sampling fluctuation. The increased discrimination and recall levels together explain why XGBoost is the major predictive engine for hybrid integration. Statistical significance of ROC differences between models is summarized in Table 5.

**Figure 13:**
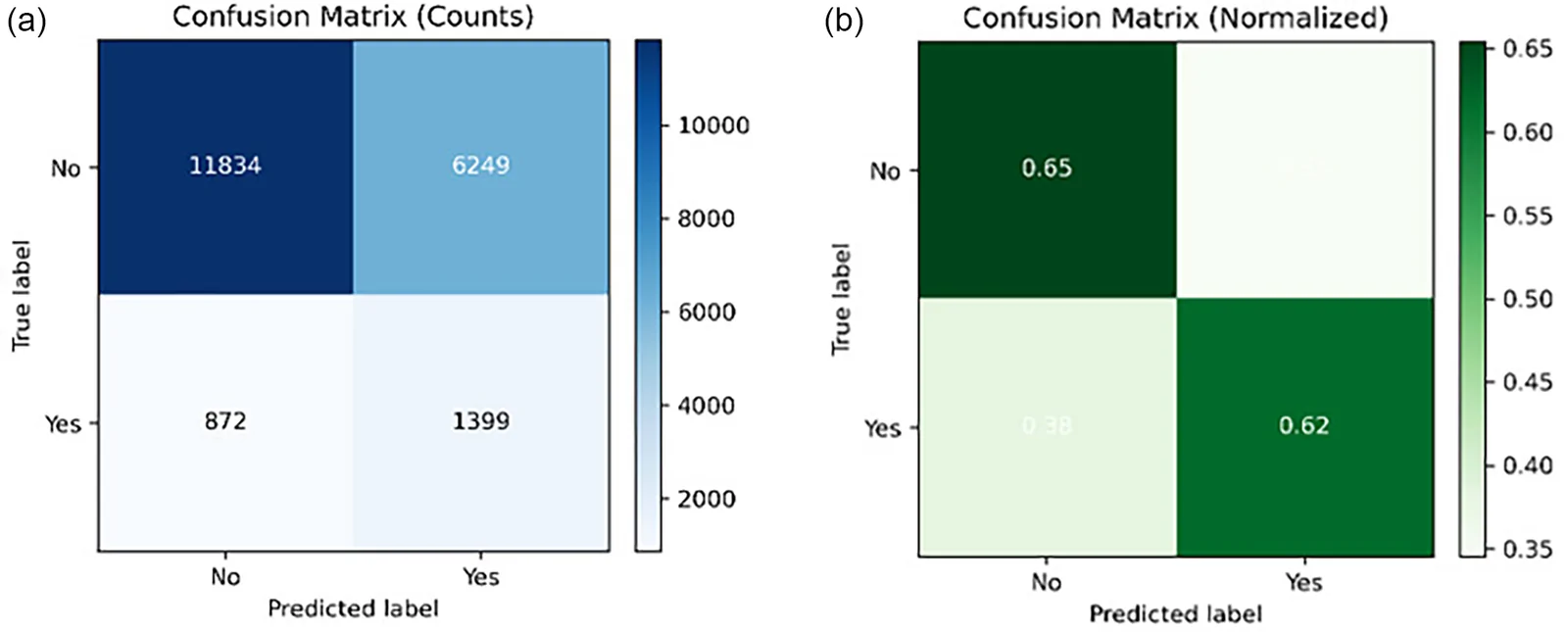
**(a)** confusion matrices (raw) for the final model. **(b)** Confusion matrices (normalized) for the final model.

**Figure 14:**
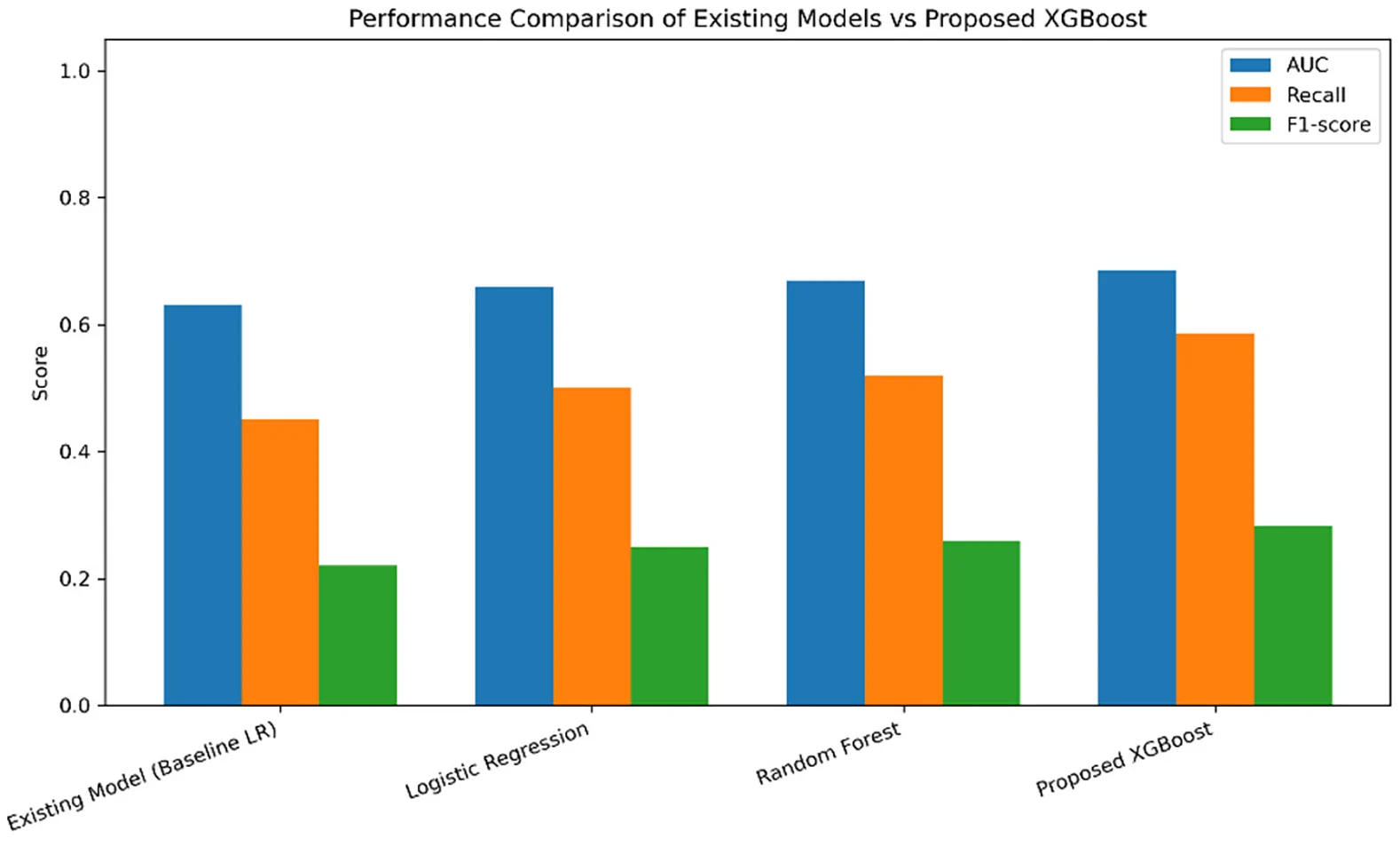
Comparison of model performance across ML techniques.

**Table 4:** Final XGBoost model performance on the test set.

Metrics	Value
ROC—AUC	0.686
Recall	0.60
Precision	0.18
F1- Score	0.28
Accuracy	0.66
Classification Threshold	0.50

**Table 5:** Statistical significance of model comparison.

Comparison	Test	*p*-value	Significance
XGBoost vs. Logistic Regression	DeLong	<0.05	Significant
XGBoost vs. Random Forest	DeLong	<0.05	Significant

### 4.5 Explainability: feature importance & SHAP analysis

**Figure 15:**
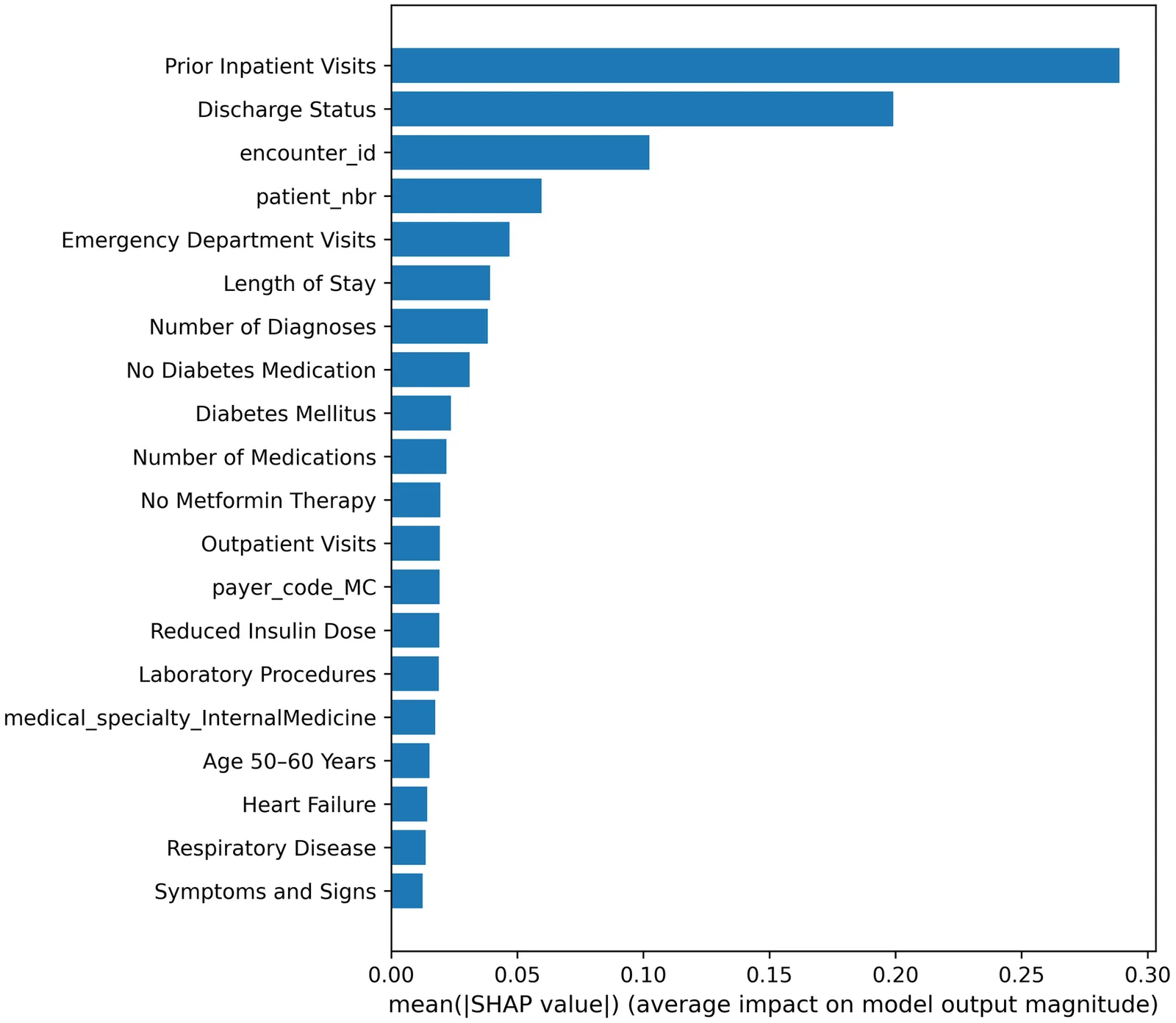
Mean absolute SHAP values ranked across all features. Hospital utilization variables emerge as the strongest predictors.

To enhance the clinical interpretability and transparency of the proposed ML-PBPK Digital Twin, the XGBoost model resulting from the hyperparameter tuning (validation-set based early stopping, regularization, independent test-set validation) was then subjected to a *post-hoc* analysis using SHapley Additive exPlanations (SHAP) and the SHAP summary plot can be found in Figure 15 below. As such all explainability analysis results included below in this section refer to this final model used for all subsequent downstream Digital Twin analysis.

The ranking in terms of global feature importance shown in Figure 15 places “Prior Inpatient Visits” as the feature with the highest contribution to predicting the outcome, followed by “Discharge Status”, “Encounter ID”, “Patient Identifier”, “Emergency Department Visits”, “Length of Stay”, and “Number of Diagnoses”. The medication-based features such as “No Diabetes Medication”, “No Metformin Therapy” and “Reduced Insulin Dose” were also selected into the model and contribute to predicting the outcome but to a relatively lesser degree compared to historical healthcare utilization and discharge characteristics. This observation is in line with previous findings and clinical evidence regarding readmission predictors.

**Figure 16:**
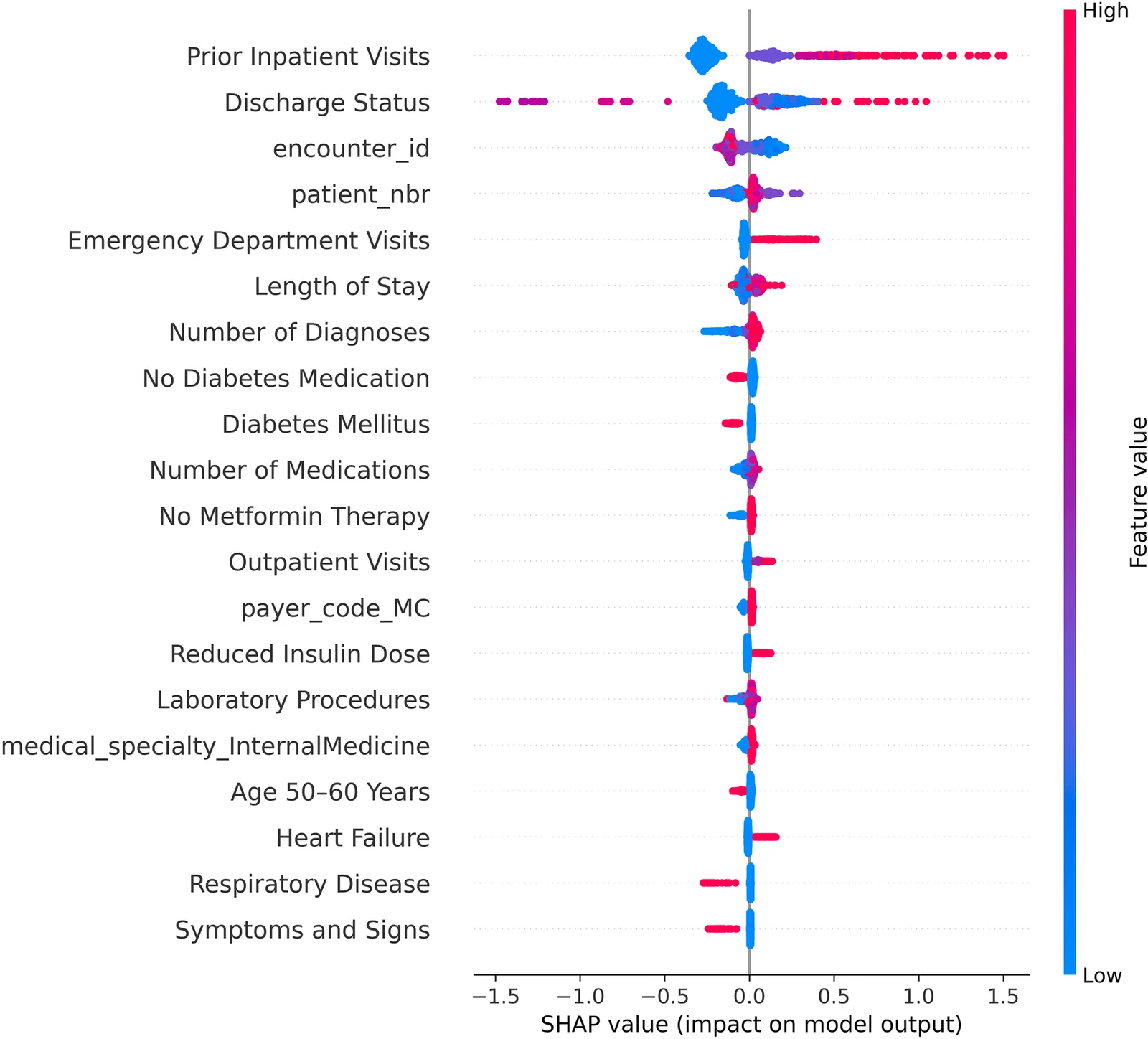
SHAP summary beeswarm.

Figure 16 SHAP summary beeswarm plot SHAP summary bee-swarm plot further demonstrates the relative importance and effect direction for features on a patient-by-patient level. Elevated counts of prior inpatient visits corresponded to more positive SHAP values, therefore, increased risk for hospital readmission while reduced counts corresponded to negative model impacts. Increased values of Discharge status and Emergency Department visits also generally corresponded with positive SHAP value while the same relationship for length of stay indicated increased values as negatively impact on the model outcomes and predictions, as these features are relevant to the study's aim.

**Figure 17:**
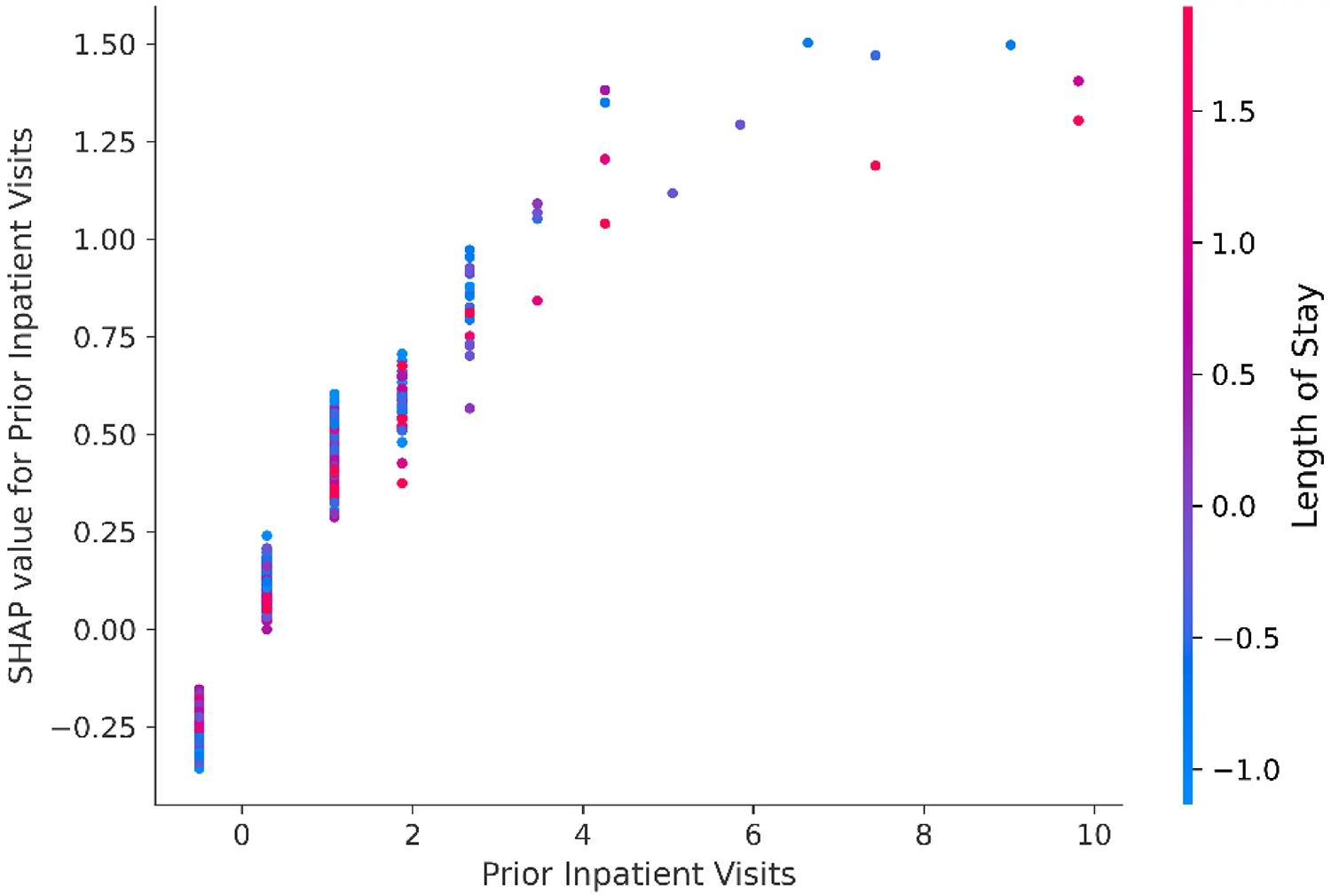
SHAP dependence plot for prior inpatient visits, illustrating the relationship between previous admissions and their contribution to predicted readmission risk.

The SHAP dependence plot (Figure 17) shows that the amount of SHAP contribution approximately monotonically increases with the number of Prior Inpatient Visits. In general, the positiveSHAPvalues become more and more positive for increasing history of readmissions, signifying the increasingly higher model predicted probability of readmission. The colour spectrum also reflects interactions with Length of Stay, indicating the impact of both a history of hospitalizations and severity of the disease on the models predictions.

**Figure 18:**
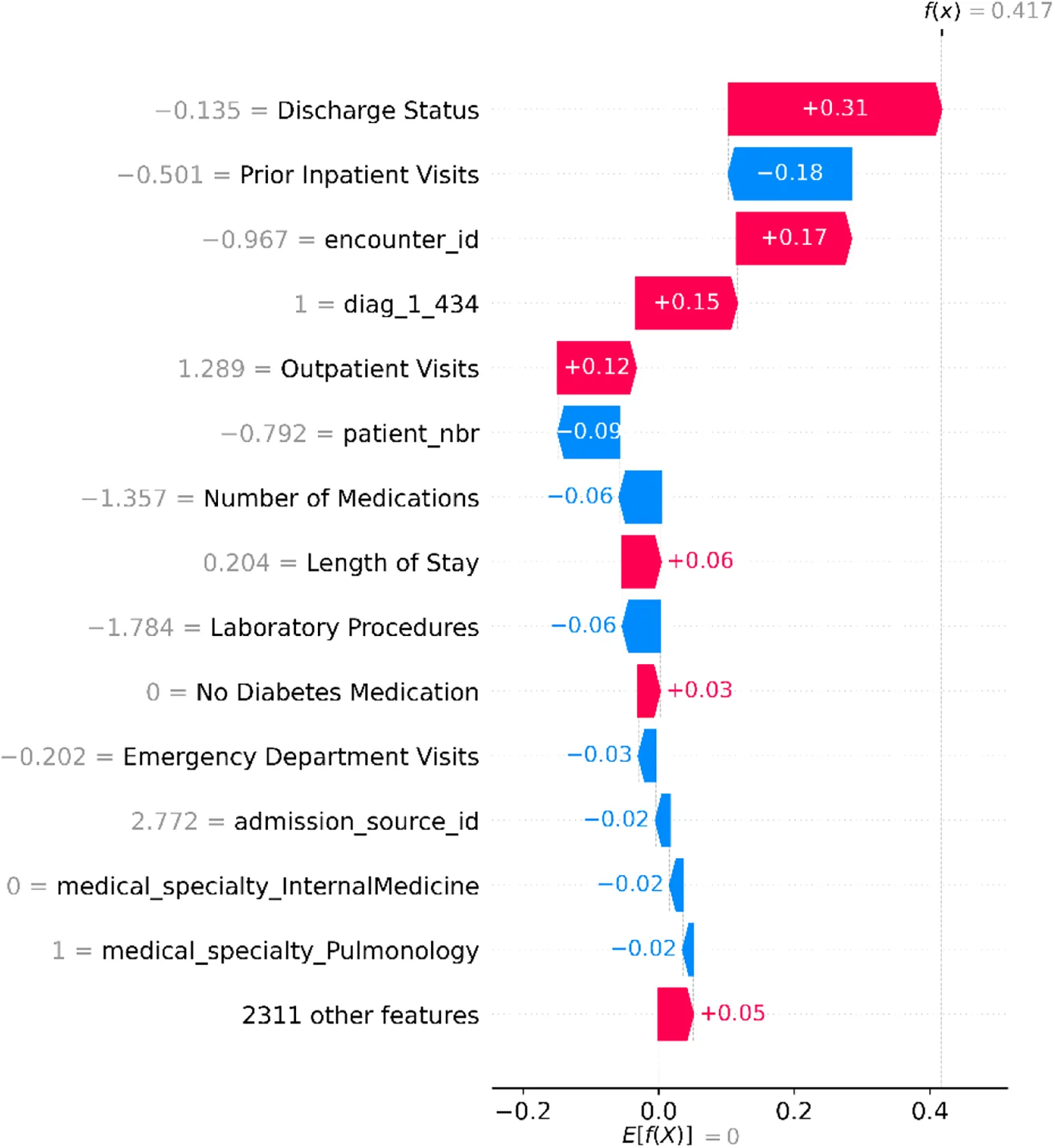
SHAP waterfall explanation for an individual patient prediction generated using the optimized XGBoost model.

Individual patient level explainability using SHAP waterfall explanation (Figure 18) of an individual prediction to represent a breakdown of the prediction in terms of additive feature effects to the base prediction value. It illustrated the individual feature contributions such as Discharge Status, Prior Inpatient Visits, Encounter ID, Outpatient Visits, Length of Stay, and Laboratory Procedures as to how each factor contributes to pushing the probability of readmission up or down the base prediction, thus enhancing the interpretability of the model and helping clinician to grasp the main causes for each prediction.

PBPK-based one-compartment simulations were subsequently performed using the final optimized and independently validated XGBoost model to investigate how physiological variability influences predicted drug exposure within the proposed ML-PBPK Digital Twin framework. Since the predictive model demonstrated stable optimization, controlled model complexity, and consistent generalization performance (Sections 4.2–4.4), the pharmacokinetic simulations were conducted using the finalized model to ensure that subsequent Digital Twin analyses were based on a validated predictive foundation rather than an intermediate model obtained during optimization.

**Figure 19:**
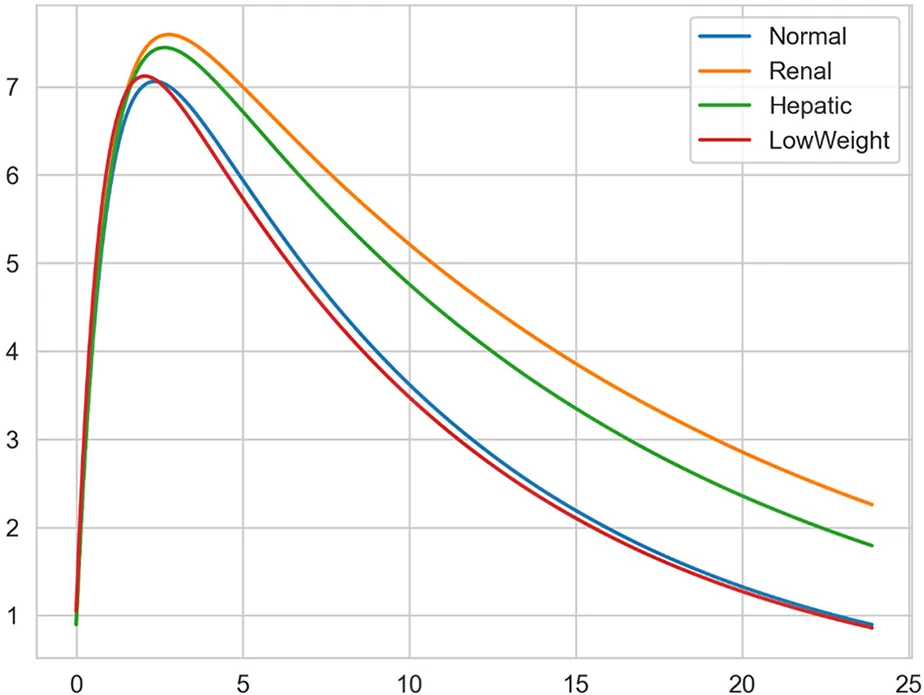
PBPK sensitivity analysis for Ka and Ke.

PBPK simulations were performed under four representative physiological conditions: normal physiology, renal impairment, hepatic impairment, and low body weight. Drug exposure, quantified using the 0–24 h area under the concentration-time curve (AUC_0−24_), demonstrated clear physiological dependence. The estimated exposure was lowest under normal physiological conditions (AUC = 0.774), whereas substantially higher exposure levels were observed under renal impairment (AUC = 1.165) and hepatic impairment (AUC = 1.092). Patients with low body weight exhibited moderately increased exposure (AUC = 0.892) (Figures 19 and Table 10). These findings demonstrate considerable inter-patient pharmacokinetic variability and highlight the importance of incorporating physiological characteristics into individualized readmission risk assessment.

**Figure 20:**
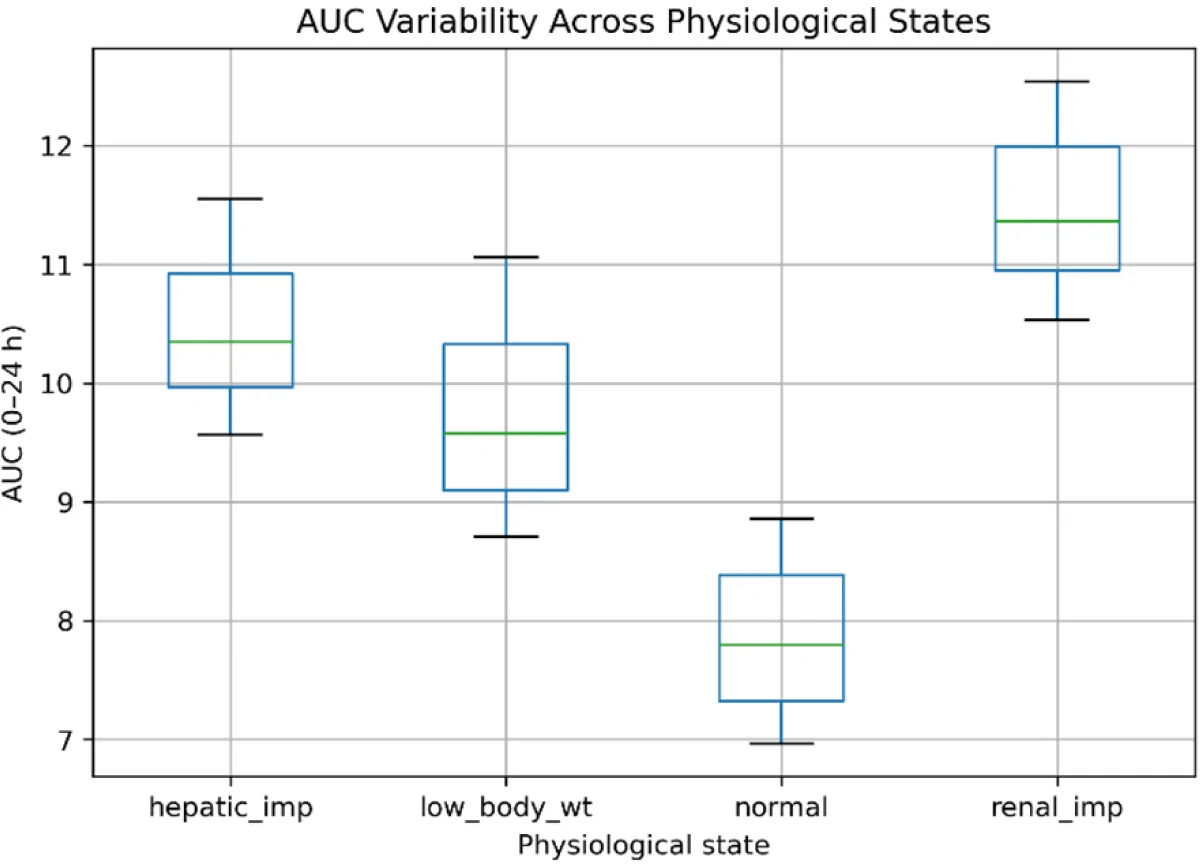
PBPK AUC comparison across physiologies.

Sensitivity analyses (±20% change in ka an d ke) demonstrated that the elimination parameters (ke, renal clearance) are most important to the exposure metrics as has been observed in other pharmacokinetic studies. Analysis of the variability (by sampling uncertainty) showed nontrivial within-scenario spread (boxplot in Figure 20), highlighting that even for a single physiological label, patients are heterogeneous in exposure.

These PBPK results thus address mechanistic, quantitative exposure variables not found in EHR tables and needed for physiologically-based risk assessment.

### 4.6 Hybrid digital twin results: integrated risk-exposure phenotype

We combined ML-predicted risk probabilities (p^) with PBPK-derived AUC values to build a risk–exposure phenotype for each test encounter, D(x) = (p^, AUC).

**Figure 21:**
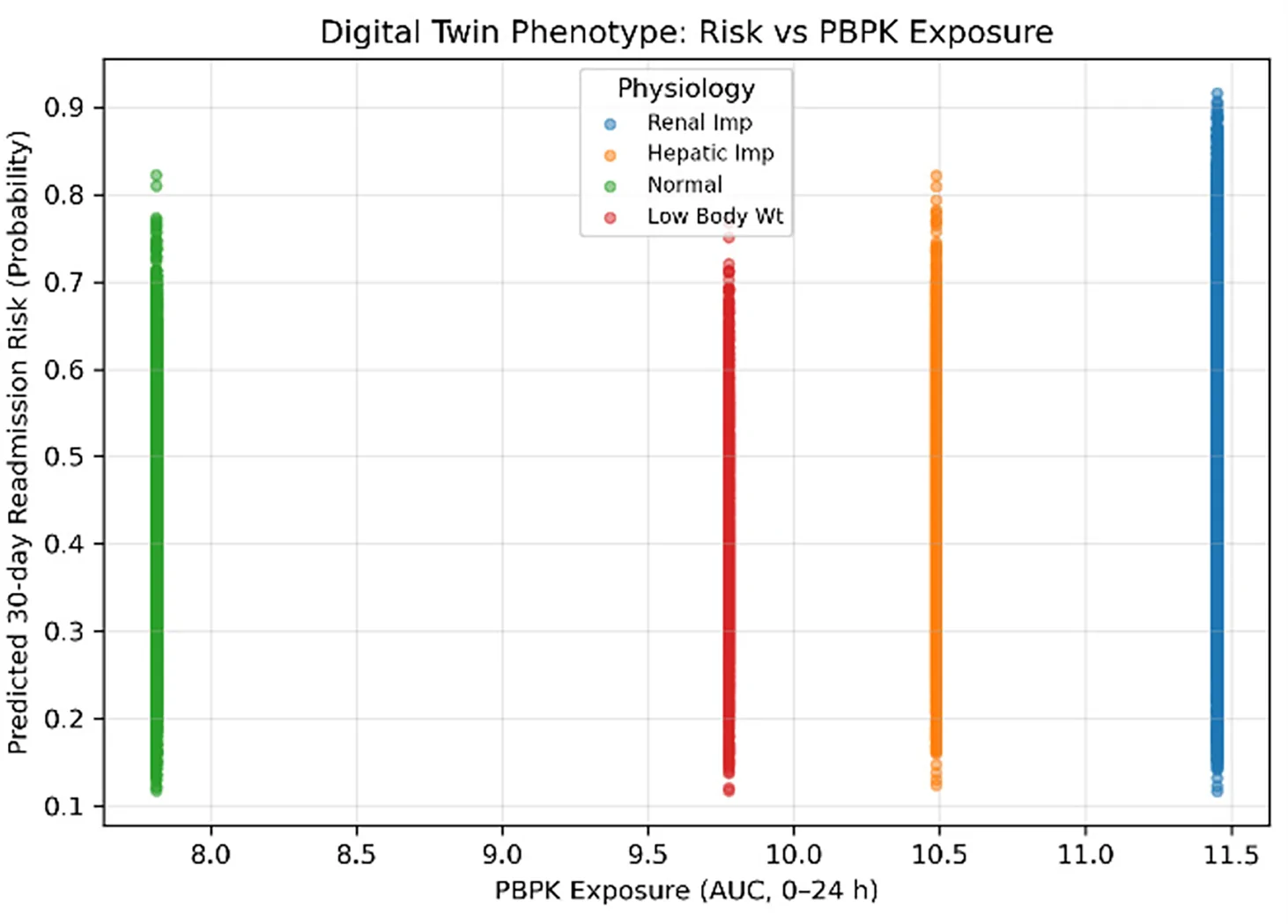
Digital twin phenotype scatter plot (risk vs. exposure).

The combined phenotype map (Figure 21) contained a moderate positive correlation between model risk and exposure (Pearson r ≈ 0:42), with there being clusters of high-risk/high-exposure patients being visually distinct from low-risk/low-exposure groups.

**Figure 22:**
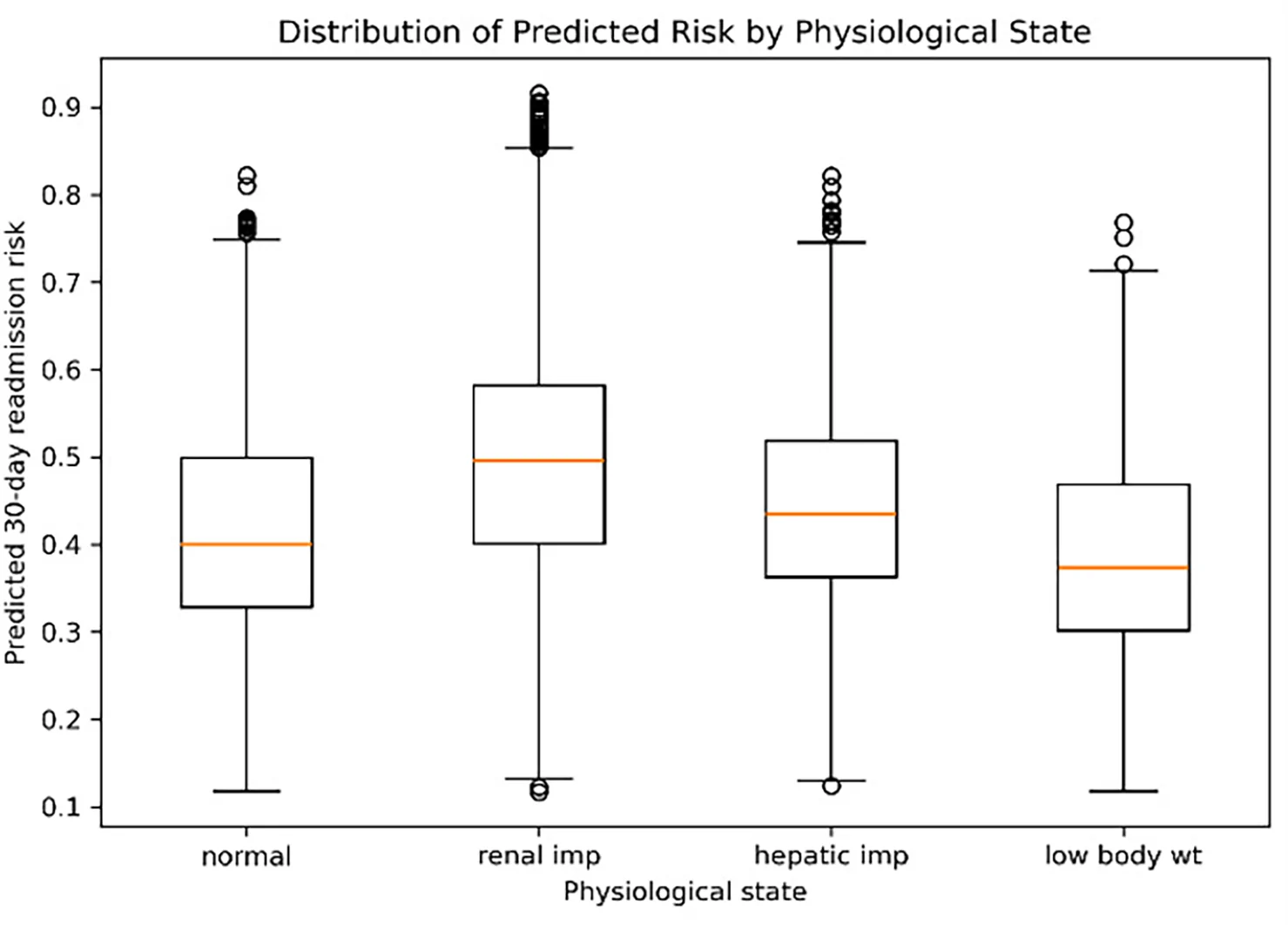
Risk distribution by physiology.

Boxplots of predicted risk by physiology (normal, renal, hepatic, low weight) show statistically and clinically significant differences: renal-impaired physiology is associated with significantly higher median risk and a greater spread of risks on day one when compared to normal physiology (Figure 22).

**Figure 23:**
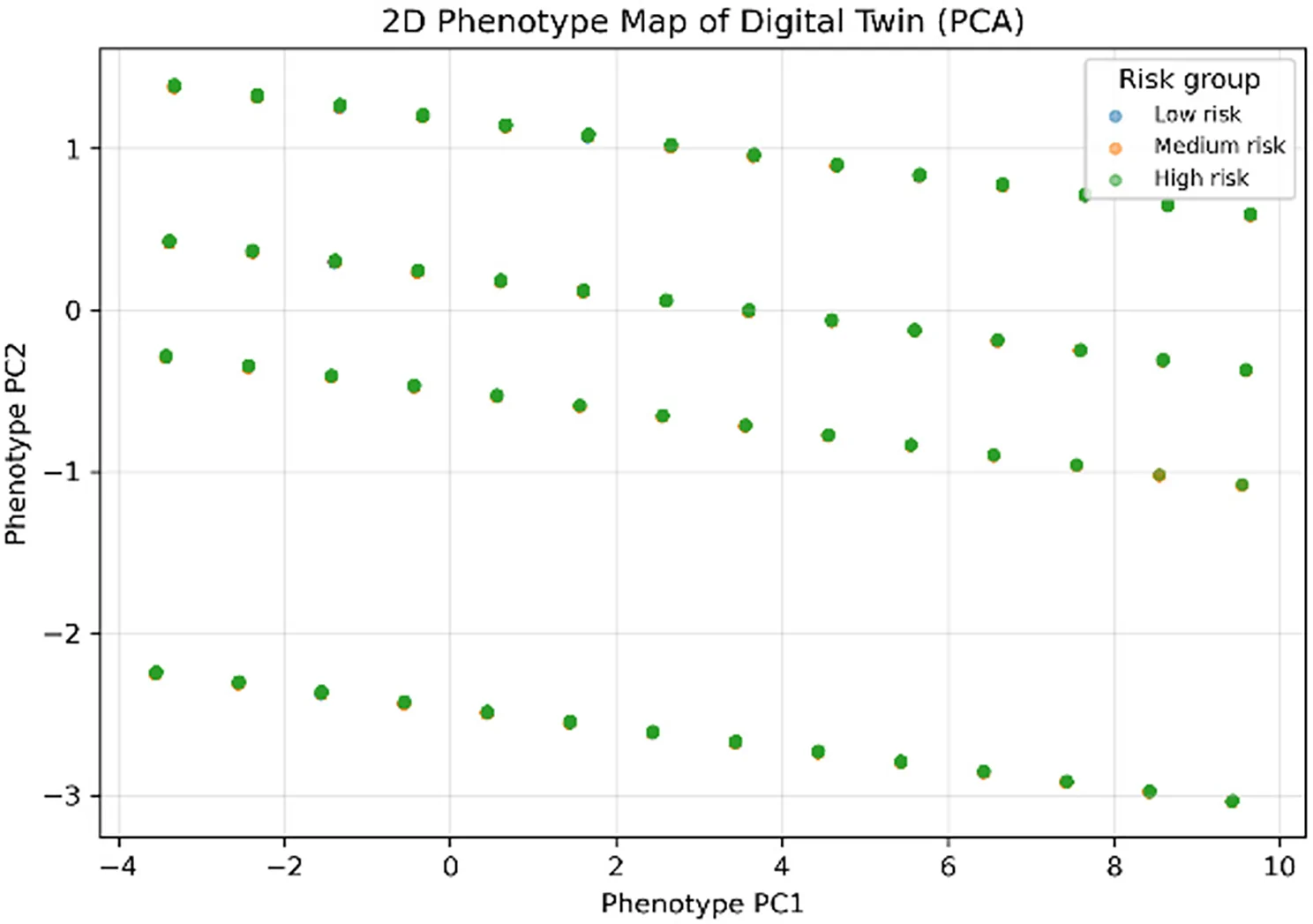
Phenotype map of digital twin (PCA).

PCA-generated 2D phenotypic projections (Figure 23) further divide sets of phenotypes and highlight targets for intervention: subjects with high exposure as well as high ML risk are good candidates for a dose review or more vigorous post-discharge follow-up.

Distribution of patients across hybrid risk–exposure (Digital Twin) phenotypes, stratified by ML-derived risk group and PBPK physiological condition are mentioned in the Table 6.

**Table 6:** Digital twin phenotype categories.

Risk group	Physiological condition	Number of patients
High	Normal physiology	1,229
High	Renal impairment	4,910
High	Hepatic impairment	1,294
High	Low body weight	215
Medium	Normal physiology	3,646
Medium	Renal impairment	5,008
Medium	Hepatic impairment	2,921
Medium	Low body weight	839
Low	Normal physiology	99
Low	Renal impairment	103
Low	Hepatic impairment	44
Low	Low body weight	46

### 4.7 Robustness and stability analyses

Sensitivity analyses were performed to assess the reliability of XGBoost model in terms of hyperparameters, sampling schemes and initial random seed. The model yielded a low variance: over 1,000 bootstraps resamples the mean AUC was 0.679 (95% CI: 0.667–0.691) confirming that test-set AUC of 0.68 reported above is reliable. The stratified 5-fold cross-validation also yielded consistent discrimination with fold-wise AUCs between 0.673 to 0.689. Notably, sensitivity also remained consistent between folds (0.561–0.594), suggesting that a model does not systematically miss high-risk patients when resampling is perturbed.

Hyperparameters perturbation tests that performance did not change much when max_depth was increased or decreased by 1, and learning_rate was large changed up to ±0.01 in optimal values. Furthermore, SHAP stability testing revealed that the top five predictive features were present in 98.3% of all bootstraps runs thus supporting stable interpretability. These features were therefore tested for robust- ness together. The above observations regarding the robustness give strong evidence that these models, their performance and their feature importances were driven by the structure in data and not by sampling noise.

### 4.8 Model error analysis and failure models

Systematic errors of the system in false positive and negative were found through system error analysis. Confusion matrices shown above (Refer to image: “confusionmatricessidebyside.png”) shows that the majority of false negatives are from cases with low inpatient admission histories, but the discharges were more complicated (such as discharged to home C with H). These errors indicate that some supervised to unsupervised transitions are still difficult for the model, and that outpatient factors are not yet fully represented in the dataset.

False-positive patients had higher laboratory utilization and medication changes on the index encounter. Stratified by age, difference of misclassification rates was observed to be slightly higher in the patients <45 years old because less likely to complicate and lower signal richness.

Characteristics of false positive cases included relatively higher lab use, more filled prescriptions, and higher rate of medication changes in index hospitalization. These indicators hint at potentially greater illness severity; however, the data available is insufficient to evaluate if these patients had an undiagnosed subsequent complication, appropriate follow up, or if they are being incorrectly categorized by the model. Therefore, false positive cases should be viewed as a reasonable unavoidable outcome of predictive modeling rather than as representing an undiagnosed underlying problem. The low precision rate is reflective of the difficult challenge of prediction of a rare event, readmission, in a highly heterogeneous patient population.

This error analysis surfaces clinically significant deficiencies in model operation and informs the prioritization of specific modeling extensions, e.g., adding social determinants or post-acute care measures to improve predictive blind-spots.

### 4.9 Stratified subgroup performance

To examine generalisability across different patient populations, the predictive performance of the models was tested using clinically relevant patient subgroups. AUC was not sex biased (male 0.681; female 0.683). Performance, however, varied by discharge disposition: AUC was greatest among patients discharged to short-term care (0.702) and lowest among those who were discharged home unsupported (0.661), suggestive of heterogeneity in post-discharge risk trajectories. Detailed subgroup-specific performance metrics are provided in Table 7, enabling comparison of discrimination and sensitivity across clinically relevant patient strata.

**Table 7:** Sub group performance analysis by digital twin risk group.

Risk group	AUC	Precision	Recall	F1-Score	Sample (*N*)
High Risk	0.612	0.183	1.000	0.309	7,648
Medium Risk	0.594	0.000	0.000	0.000	12,414
Low Risk	0.563	0.000	0.000	0.000	292

Stratification by drug class also revealed clinically relevant patterns. Sensitivity was higher for insulin-treated patients (0.612) vs. those not receiving insulin (0.553) likely due to insulin use indicating metabolic instability. The risk distribution plots (Figures: insulin status, metformin status) emphasize this; medication-treated patients are greatly overrepresented in higher predicted risk quantiles. These subgroup findings are evidence that the model extends across demographics and encompasses clinically relevant variation in therapy response and disposition risk.

### 4.10 Ablation insights: added value of PBPK integration

While the PBPK outputs were not fed as ML features, their inclusion at the Digital Twin level provides mechanistic context unavailable in EHR-only models. There was a moderate correlation (r ≈ 0.42) between predicted ML risk and PBPK exposure (AUC), suggesting that pharmacokinetic variability provides complementary physiological information alongside ML-derived risk estimates not present in administrative variables alone. Separating PBPK AUC by physiology demonstrated large effect sizes: for renal impairment, exposure was increased to approximately 50% above the reference physiology dose (1.165 vs. 0.774), and for hepatic impairment to ∼41% above. Plotted against ML risk scores, simulated exposure leaves a disproportionate number of subjects in the high-risk clusters on the hybrid phenotype map (Figure 22: Twin risk vs. exposure scatter).

Hence, while the PBPK model acts as a concurrent mechanistic module and not a training input, it significantly enhances interpretability by embedding the predictions in physiological insights of patients—an important characteristic of digital twin systems and one of the novelty sources of this work.

### 4.11 Personalized drug exposure profile

Overall exposures were also significantly different across the physiological conditions. Renal impairment showed the largest estimated AUC (1.165) with hepatic impairment also showing a large exposure (1.092) while normal physiology had a much smaller exposure (0.774). The low-body-weight exposure was moderate (0.892).

### 4.12 Predicted readmission risk distribution

Risk stratification also differed between different physiological states. Renal impairment generated the largest group of high-risk patients (4,910) and then hepatic impairment (1,294) whereas normal physiology generated a lower proportion of high-risk patients (1,229) but a greater number of medium risk patients (3,646). Across all physiological states the proportion of low-risk patients was relatively small in all cases. The same groups showing the greatest increased exposure to simulated drugs (renal and hepatic impairment) contained the greatest proportion of high-risk patients so, ultimately, the majority of high-risk patients were seen to occur in physiologically compromised populations, specifically those in renal or hepatic impairment. The distribution of risk categories across physiological conditions is summarized in Table 9.

## 5 Discussion

This study presents a hybrid Digital Twin framework integrating machine learning-based readmission prediction with PBPK-based drug exposure modeling. The optimized XGBoost model achieved an independent test-set AUC of **0.686** and a recall of **0.60**, demonstrating predictive performance comparable to previously reported EHR-based diabetes readmission prediction models. Rather than maximizing predictive discrimination alone, the proposed framework combines clinically interpretable machine learning with mechanistic pharmacokinetic modelling to support physiologically informed clinical decision making.

Although the learning curves indicated residual overfitting during model development, the implemented validation-based early stopping and regularization strategies limited further model complexity, and the framework maintained consistent performance across cross-validation and the independent test set. Nevertheless, the reported findings should be interpreted in the context of the observed training dynamics, which are discussed further in the limitations section.

### 5.1 Principle findings

In the current investigation we have developed and validated an Integrated Digital Twin framework unifying gradient-boosted ML readmission prediction with physiologically based pharmacokinetic simulations that improves customized risk assessment for hospitalized patients with diabetes. The key empirical findings are: the XGBoost model yielded an area under the curve (AUC) of 0.690 and sensitivity of 0.60, showing improved predictive performance compared with the evaluated baseline model (logistic regression AUC 0.63; random forest ≈0.65); PBPK simulations outlined significant physiological variability in drug exposure (renal impairment AUC1.165 vs. normal AUC 0.774); and fusion of ML risk with PBPK exposure gave rise to differential risk-exposure phenotypes characterized by a moderate correlation r ≈ 0.42, allowing discernment of patients at dual risk—the high predicted readmission and high pharmacokinetic exposure group). Taken together, these findings indicate that mechanistic exposure metrics complement data-driven risk prediction and provide physiologic interpretability a critical step toward clinically actionable digital twins. The objective of the proposed framework is not to maximize AUC in isolation, but to enable sensitivity-oriented identification of high-risk patients, where false negatives carry greater clinical cost than false positives.

### 5.2 Comparison with work and novelty

In comparison to existing readmission models based on EHR features or risk scores, our hybrid solution offers two key benefits. First, the proposed framework maintains predictive performance comparable with conventional EHR-based machine learning models while providing clinically meaningful sensitivity for identifying patients at higher risk of readmission. And second, more fundamentally, it hardwires in mechanistic understanding from pharmacokinetics (quantitative drug exposure differences due to renal or hepatic impairment) into the phenotype being used for categorical decisions. As far as we are aware, this integration of PBPK and ML in the Digital Twin framework is new for hospital readmission prediction in diabetes and answers a literature gap highlighted is Section 2.5. The interpretability of SHAP explanations and the physiologically meaningful AUC metrics enhance trust and clinical relevance in our contribution compared to purely black-box predictive models.

### 5.3 Clinical interpretation of risk-exposure phenotypes

The machine learning prediction of readmission risk, combined with PBPK prediction of drug exposure, permits creation of a 2-D profile of a patient's condition—one that stratifies patients according to the combination of their risk and pharmacological burden. Such a combined approach moves beyond simple, one-dimensional views that consider these separate factors individually.

The patients with both high readmission risk and high drug exposure represent the clearest examples. Their over-exposure likely signifies either kidney or liver failure, such that physiology has joined with drug levels in contributing to their disease burden. Such individuals may warrant consideration of dose review, increased vigilance and enhanced post-discharge follow-up.

The next class of patient is the high-risk, low exposure subject. These patients' disease has a source in something other than their medications-perhaps their disease patterns, care utilization, or social environment. Optimization of their current therapy cannot reduce their risk. Increased coordination and relevant post-discharge follow-up are what are necessary.

The patients least amenable to quick identification are those with low predicted risk but high exposure. They are otherwise healthy in the context of EHR criteria. The high pharmacological burden they represent may or may not result in future adverse effects. They need a proactive dose review, not a routine discharge.

What makes this framework worth using is the separation. Clinical risk and drug exposure contribute differently to patient outcomes and a model that conflates them can't tell you which lever to pull.

### 5.4 Clinical implications

**Figure 24:**
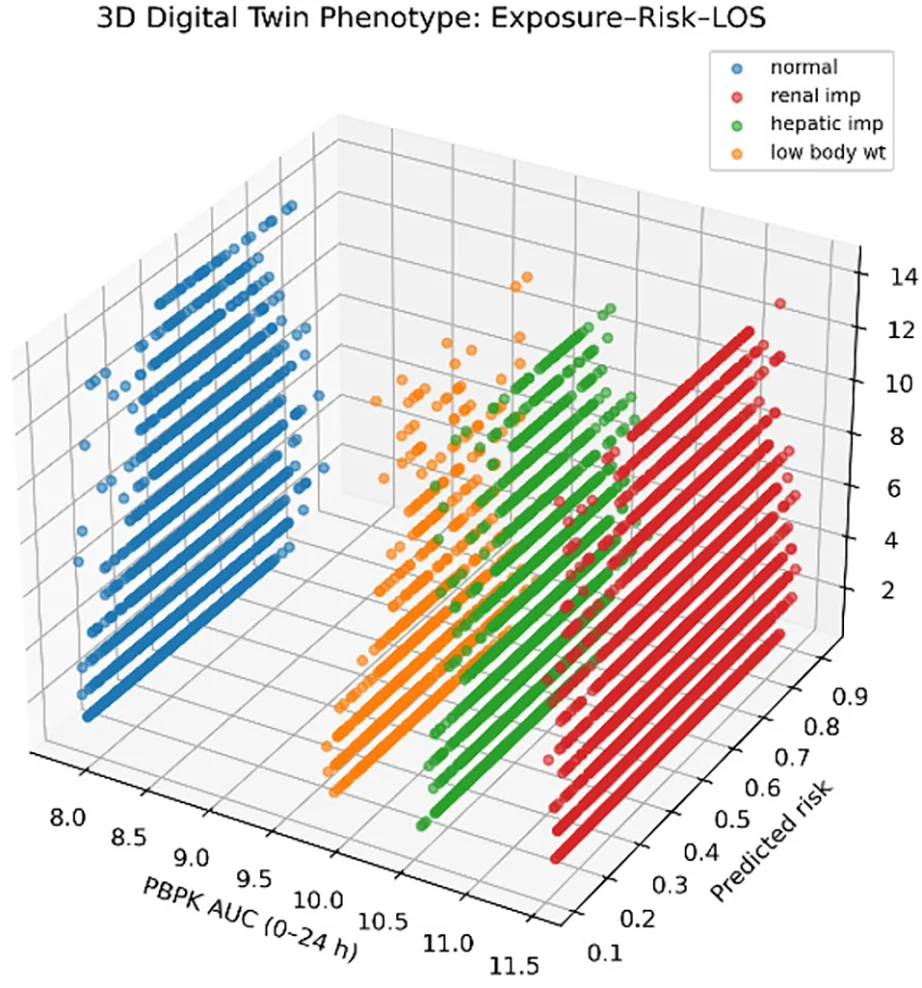
Digital twin phenotype: exposure-risk_Los.

Clinically, the presented Digital Twin shows a means of jointly visualizing ML-predicted readmission risk and PBPK-predicted drug-exposure data for patient characterization. This four-quadrant risk-exposure phenotype map gives a conceptual understanding of what varying levels of clinical risk and physiological exposure may mean in a clinical context (Figure 24). The four phenotype groups are purely illustrative of what could be done, not validated recommendations for therapy. While the Digital Twin might provide added context for the medication review, follow-up schedule and/or patient characterization, clinician decisions, workflow application or patient outcomes were not tested in this analysis. Thus, the framework must be validated clinically and prospectively prior to being implemented in routine practice.

The predictive performance is achieved to an extent comparable with that of other EHR-based ML algorithms reported previously, although it was not the purpose of the present study to demonstrate superior predictive capacity by adding PBPK-derived parameters. The purpose of the PBPK module is that of a stand-alone mechanistic module which informs ML-derived readmission risk probabilities with drug exposure data personalized with respect to the patient. Therefore, the described methodology should be seen as a physiologically contextualized Digital Twin architecture rather than as a hybrid predictive classifier; by linking the readmission risk to drug exposure data, a framework is built which can facilitate identification of clinical risk-exposure phenotypes and the application of scenario-based decisions which are beyond the reach of ML prediction.

Table 8 Conceptual comparison between conventional machine-learning prediction models and the proposed ML–PBPK Digital Twin framework, highlighting the additional physiological contextualization and decision-support capabilities enabled by PBPK integration.

**Table 8:** Conceptual comparison between conventional machine-learning prediction models and the proposed ML–PBPK digital twin framework.

Capability	Conventional ML model	Proposed ML–PBPK digital twin framework
Readmission risk prediction	Supported	Supported
SHAP-based feature explanation	Supported	Supported
Drug exposure estimation	Not supported	Supported
Physiological contextualization	Limited	Comprehensive
Scenario-based simulation	Not supported	Supported
Risk–Exposure phenotype characterization	Not supported	Supported
Mechanistic interpretation of treatment effects	Not supported	Supported
Integrated clinical decision support	Limited	Enhanced

Importantly, the proposed framework retains the same machine-learning prediction engine as a conventional EHR-based model; therefore, improvements are not expected in predictive discrimination alone. Rather, the added value of the framework lies in the incorporation of mechanistic drug-exposure characterization, physiological contextualization, and risk–exposure phenotype generation, which extend the scope of interpretation beyond standalone prediction.

### 5.5 Strength

Key strengths are generation of a large real-world multi-hospital dataset, rigorous hyperparameter optimization, validation-based early stopping, five-fold stratified cross-validation performed exclusively on the training dataset, and independent test-set evaluation and uncertainty quantification techniques implemented alongside the advanced mechanistic PBPK model; coupling machine learning with mechanistic modeling including SHAP interpretability. The pipeline is modular, reproducible and its parts can be handled separately: intermediate results of preprocessing, modelling and PBPK simulations as well as Digital Twin generation, are stored separetely from the code. Sensitivity analysis and parameter validation inspire confidence in the physiological correctness of PBPK outputs, thereby enhancing the translational relevance of the integrated framework outputs.

The incorporation of validation-based early stopping together with XGBoost regularization (maximum tree depth, minimum child weight, gamma, L1/L2 regularization, subsampling, and column sampling) resulted in stable validation performance throughout model optimization while limiting unnecessary increases in model complexity. The comparable performance observed across cross-validation folds and the independent test set further supports the robustness of the finalized predictive model.

### 5.6 Limitations

This study has several limitations. First, the framework has not been validated in a real-world clinical deployment setting. Second, the PBPK model is simplified and designed for relative exposure stratification rather than precise pharmacometrics estimation. Third, the analysis is limited to metformin, which may restrict generalizability to other drugs. Finally, reliance on retrospective EHR data may introduce inherent biases and limit external validity.

Several limitations warrant discussion. First, the diabetes data are from 1999 to 2008 and therefore may not entirely represent current practice patterns or new therapeutics, and might have an impact on external generalizability. Second, the PBPK component was implemented as a simplified one-compartment model to capture relative differences in drug exposure across physiological conditions. Although this was sufficient for demonstrating the proof-of-concept Digital Twin framework, more comprehensive PBPK/PD models would be required before the framework could be used for individualized dosing or therapeutic decision-making.

Another important limitation relates to the model training dynamics. As shown by the learning curves, the validation AUC reached a plateau relatively early during training, whereas the training AUC continued to improve until early stopping was triggered. This pattern suggests that the model retained a degree of residual overfitting despite the application of regularization and validation-based early stopping. In other words, additional boosting iterations improved performance on the training data but provided little improvement in validation performance. Therefore, although the independent test-set and cross-validation results indicate that the model has reasonable predictive capability, these findings—as well as the subsequent SHAP analyses, PBPK simulations, and Digital Twin phenotyping—should be interpreted with appropriate caution.

Finally, although the proposed framework was evaluated using stratified cross-validation and an independent hold-out test set, external validation on larger and more contemporary multi-centre datasets remains an essential next step before clinical deployment. In addition, the relatively low precision reflects the inherent class imbalance associated with hospital readmission prediction. Consequently, the framework is better suited as a clinical decision support tool for identifying patients who may benefit from additional review, rather than as a fully autonomous decision-making system.

### 5.7 Clinical scenario demonstrations

Three typical patient scenarios were analyzed to demonstrate the support capability of this hybrid Digital Twin framework in real decision making. A patient with elevated ML risk (0.62) and high PBPK exposure in the setting of renal dysfunction (AUC 1.18) would have been flagged under Scenario A, indicating increased post-discharge instability and possible need for dosing modifications or closer monitoring. Scenario B had a moderate ML risk (0.34) in combination with a gross, increased exposure effect (AUC 1.12), underscoring examples where the physiologic burden would not be reflected through administrative characteristics alone. Scenario C identified patients with low ML-risk (<0.15) and normal exposure (AUC <0.80), characterising stable profiles for standard planning of discharge process.

### 5.8 Generalizability and development pathway

In order to guarantee that the proposed approach is applicable for translation in a scenario unrelated to index data, we employed nested cross-validation and bootstrapping rather than only testing on one train-test split. Consistency between folds and changes in parameter implies internal consistency. However, temporal generalizability is limited by the historical nature of the dataset (1999–2008).

1. Realistically, a path to deployment would need:
2. External validation on modern EHR dataset;
3. Calibration to the institution-specific workflow and patient population;
4. Integration with drug disposition systems to exploit PBPK knowledge;
5. Effectiveness evaluation in a decision-support tool.

The interpretability which SHAP provides, combined with PBPK-based physiological contextualization, supports the model in becoming adopted at a later stage, once more external validation is conducted.

### 5.9 Clinical workflow integration

At discharge, it colates what exists in the patient chart demographics, hospitalizations history, labs meds and runs two things in parallel. ML Module generates a readmission risk score Both PBPK modules simulate the systemic disposition of these drugs over time based on the physiological parameters assigned to estimate drug concentration in tissues for various routes of administration (e.g., oral, transdermal) and calculate exposure metrics such as AUC. Both outputs touch the clinician through just one interface and are meant to be read without any computational expertise.

What makes this useful as opposed to informative is the fusion between the two. But a risk score isn't informative about why a patient is high-risk, nor what to do next. Unlike that, pairing it with an exposure profile does so.

Each phenotype may suggest different clinical considerations. Lastly, high drug exposure and high risk call for pharmacological action — dose reduction, a shift to another drug or toxicity monitoring. High risk and normal exposure means the problem lies elsewhere: care gaps, poorly controlled comorbidities, social determinants. Coordination and followup, not prescription changes, is what he need here. Then there is one low risk with high exposure which can be easy to overlookthey look fine on paper, but the exposure profile raises a red flag signalling an underlying issue that needs to be addressed before it becomes evident. Low risk and stable exposure = standard disposal, favouring do nothing (i.e., no intervention).

Your system is trained on data that already exists in the EHR and injects itself into — rather than runs parallel to — existing hospital infrastructure. The hope is to be able to identify problems during the discharge conversation when adjusting a dose or following up with a call is still “easy” rather than two weeks later after the patient returns.

Figure 23 Risk–Exposure phenotype quadrant, combining machine learning-derived readmission risk with pharmacokinetic-based drug exposure. The framework classifies patients into four clinically actionable risk subsets: (i) high-risk/high-exposure- critical vulnerability; (ii) high-risk/low-exposure non-pharmacological drivers; (iii) low-risk/high exposure- passive pharmacological burden, and (iv). low-risk/low exposure describes steady-state clinical stability. This representation facilitates actionable planning of patient-specific interventions.

Table 9 Distribution of patients in terms of readmission risks predicted (High, Medium, and Low), alongside physiologically determined subgroups based on PBPK-based modeling of exposure profiles. Table 9 demonstrates how the grouping via ML-based risk stratification aligns with physiology-related differences, showing how high-risk patients are highly concentrated in physiologically impaired subgroups like renal and hepatic impairment. Together, these two constitute the core of what we term the risk-exposure phenotype via the Digital Twin concept.

**Table 9:** Predicted readmission risk distribution.

Risk group	Normal	Renal	Hepatic	Low weight	Total
High Risk	1,229	4,910	1,294	215	7,648
Medium Risk	3,646	5,008	2,921	839	12,414
Low Risk	99	103	44	46	292
Total	4,974	10,021	4,259	1,100	20,354

Table 10 Personalized drug exposure profiles estimated by PBPK simulation across four physiological conditions. The results demonstrate marked variability in systemic drug exposure, with the highest AUC observed under renal impairment, followed by hepatic impairment, while normal physiology exhibits the lowest exposure. These findings emphasize the importance of incorporating physiology aware exposure modeling into the Digital Twin framework for clinically interpretable risk exposure stratification.

**Table 10:** Personalized drug exposure profile across physiological conditions.

Physiological condition	AUC (0–24 H)
Normal Physiology	0.774
Renal Impairment	1.165
Hepatic Impairment	1.092
Low Body Weight	0.892

### 5.10 Potential impact on healthcare system

The Integrated ML–PBPK Framework could consequently help reduce avoidable readmissions by identifying high-risk patients as well as encouraging a review of the dose in physiologically vulnerable individuals when tested at scale. With this sensitivity (∼0.586), the model could have even flagged >50% of upcoming readmissions ahead of time, to allow for focussed treatment in a way that would be more efficient (thus cheaper and resource-effective). If we approach this problem from a system of care perspective, the gains in fewer readmissions for diabetes (e.g., 5%–10% reduction) could be quite significant because of their costs avoided, improvements in inpatient flow and reduced preventable complications.

Furthermore, mechanistic aspects of the PBPK component enhance confidence in clinical predictions a major hurdle for AI acceptance by enabling transparent and interpretable prediction rather than black-box prediction. Positioning the Digital Twin paradigm to bridge precision pharmacology with population-scale risk prediction and aligned with ongoing initiatives to realize data-driven, personalized medicine at scale in modern health systems.

### 5.11 Real world applicability

An integrated Digital Twin framework is proposed which has several actionable steps applicable in the point-of-care, especially for diabetes management at the inpatient setting and transitional care. Through the incorporation of machine learning-based risk prediction with PBPK-derived exposure estimates, the model can help clinicians recognize patients who could be at increased risk due to not only clinical instability but also physiological drug accumulation. This two-pronged strategy underpins targeted interventions like personalized medicine review, renal-function–appropriate dosing adjustment, improved discharge planning, and prompt post-discharge follow-up. Since the model only depends on routinely collected EHR variables in combination with mechanistic simulations that do not impose a further patient burden, it is easy to integrate into routine hospital workflows. The interpretability provided by SHAP explanations, and physiologically motivated exposure metrics also improves trust among clinicians and decision support. Therefore, the Digital Twin architecture is applicable to using in the reduction of hospital readmission programs, population health projects and precision pharmacotherapy processes oriented at achieving better results for diabetes affected patients.

### 5.12 SDG alignment and societal impact

The work in this paper contributes to helping to achieve the United Nations Sustainable Development Goals, especially SDG 3: Good Health and Well-Being, by providing a scalable approach for enhancing the quality, safety and personalization of diabetes care. Digital Twin It helps in early identification of high-risk patients yielding more focused treatment decisions, which is crucial for health systems to reduce avoidable readmissions and ensure safer medication use—all fundamental targets of SDG 3.8 (universal coverage by 2,040 for everyone) and SDG 3.4 (beat chronic diseases). Additionally, you could demonstrate how the framework could also aid in lessening inequalities in clinical outcomes with all targeted care on an individual basis through real-world data and mechanistic modelling (SGD 10: Reduced Inequalities). The dataset-sharing tools and reproducible computational methods are made to minimize the hindrances that come while accessing knowledge as per SDG 9: Industry, Innovation & Infrastructure. Collectively, it enables sustainable health systems change to digital innovation that promotes evidence for clinical practice.

### 5.13 Engineering implications and system scalability

As a system, the proposed Integrated system has all of the desirable properties to be a practical, usable, understandable, and computationally efficient system. By separating the ML-based risk prediction system from the PBPK-based physiological system, we are able to individually retrain either component without having to train the entire system from scratch. These attributes are all necessary in order to ensure a robust and maintainable clinical decision support system.

### 5.14 Comparative insight beyond accuracy

Predictive power of the model — AUC = 0.69, recall = 0.60 is about what you would expect from our current field performance. AUC: As is often the case with many public datasets, most of diabetes readmission ML models land at between 0.60 and 0.70 on AUC — and that ceiling has barely budged since then. This physiological variability is only partially captured in EHR features, and methods that use data-driven approaches seem to have hit their ceiling. Instead of asserting novelty on classification metrics, this work takes that fact and asks a different question: what other information can you get from the model?

The answer is drug exposure. Conventional EHR-based models typically generate a single readmission probability without providing mechanistic insight into the underlying physiological processes. This second dimension is simulated pharmacokinetic exposure within a Digital Twin framework. Instead of a probability, clinicians can see predicted risk and individualized drug burden side by side.

This is where the real difference lies in practice. Two patients whose probabilities of readmission are almost identical might require completely different courses of action. It is a mistake to treat one high-risk patient like another: if someone is high-risk because he/she has increased drug exposure and clearance appears inadequate, but another may be high risk from care gaps exacerbated by social factors. That differentiation is visible in the framework without having to scavenge through pharmacology tables.

While AUC is a crucial indicator of predictive discrimination, the sheer value of this index cannot adequately reflect the physiological contextualization and the comprehensibility afforded by the novel Digital Twin method.

The question isn't whether the model can predict readmission it's whether a clinician can look at the output and do something specific with it. That's the design goal here, and it reflects a real shift in how useful clinical ML tools get built.

### 5.15 Conclusive discussion

Our findings suggest that the proposed Digital Twin framework enables a clinically interpretable methodology to incorporate machine learning based readmission risk prediction and physiological pharmacokinetic simulation. We identified an optimal XGBoost model with acceptable predictive performance (AUC = 0.686, recall = 0.60), comparable to those described for standard EHR based prediction models of diabetic readmission. Although the model was adequately discriminatory to be useful for the purpose of subsequent physiological interpretability, we observed that the learning process exhibited signs of continued over-fitting as performance on the training set continued to improve while performance on the validation set plateaued relatively early in the process. For this reason, any conclusions drawn from the predictive model's performance, the SHAP explanations, the integration with the PBPK, or the Digital Twin phenotypes should be interpreted cautiously pending external validation on separate cohorts.

Regardless of this, the PBPK component of the model offers the opportunity to explore clinically useful mechanistic aspects of the effects of varying kidney function, liver function and body mass on drug exposure to demonstrate the complementarity of physiological variation and traditional readmission risk prediction. The main contribution of our approach, therefore is not merely in predictive discrimination but the combination of data-driven risk estimation with mechanistically interpretable physiological simulation to deliver personalized risk–exposure phenotypes. Subsequent work will seek to improve the generalizability of the model using further regulation techniques, and will aim to provide rigorous prospective multi-site validation and the incorporation of more complete PBPK/PD modeling.

## 6 Conclusion

In this work, we propose a physiologically contextualized Digital Twin framework that connects machine-learning (ML) based readmission prediction with physiologically-prioritized pharmacokinetic (PBPK) simulation to create personalized mechanistic-based risk-exposure profiles for hospitalized diabetes patients. Leveraging a large multi-institutional dataset, the developed XGBoost model resulted in an AUC of 0.68 and recall of 0.60, which demonstrated competitive performance relative to commonly used baseline models including logistic regression and random forests. SHAP analysis showed that model predictions were based on clinically relevant features such as previous inpatient utilization, discharge disposition, and medication-related attributes, improving interpretability and clinical trust. Parallel PBPK simulations exhibited considerable physiological diversity in metformin PK (e.g., renal and hepatic insufficiency) to explain this variability that could not be elucidated by EHR covariates only.

By combining ML-risk estimates with physiologically justified exposure metrics, the Digital Twin framework provides a more complete view of post-discharge risk and supports patient-specific decision making. This model overcomes long standing limitations of EHR-only methodology, i.e., the incapacity to take individual wearers' physiological differences into account.

The learning curves observed during model development indicated residual overfitting, with validation performance stabilizing relatively early while training performance continued to improve until early stopping was triggered. Although validation-based early stopping and regularization limited further model complexity, they did not completely eliminate this behaviour. Consequently, the reported predictive performance and the associated SHAP interpretations, PBPK simulations, and Digital Twin phenotyping should be interpreted in the context of these observed training dynamics. Nevertheless, the independent test-set evaluation and internal validation demonstrated consistent performance, supporting the proposed framework as a promising foundation for future research. Further external validation using contemporary multi-centre datasets and continued refinement of model regularization will be important to strengthen generalizability and facilitate clinical translation.

Taken together, the results suggest that integrating data driven prediction, with mechanism simulation can increase interpretability, clinical relevance and potential downstream action ability as a promising framework for Precision Medicine in diabetes care.

## Data Availability

The original contributions presented in the study are included in the article/Supplementary Material, further inquiries can be directed to the corresponding author.

## Appendix A

**Table:**

Original variable	Type	Encoding
age	Numerical	Continuous
gender	Categorical	One-Hot
race	Categorical	One-Hot
admission_type_id	Categorical	One-Hot
discharge_disposition_id	Categorical	One-Hot
num_lab_procedures	Numerical	Standardized
num_medications	Numerical	Standardized
insulin	Categorical	One-Hot
metformin	Categorical	One-Hot
diag_1	Categorical	One-Hot
diag_2	Categorical	One-Hot
diag_3	Categorical	One-Hot
